# Global, regional, and national burden of HIV and tuberculosis and predictions by Bayesian age-period-cohort analysis: a systematic analysis for the global burden of disease study 2021

**DOI:** 10.3389/frph.2024.1475498

**Published:** 2024-12-10

**Authors:** Xuebin Tian, Chong Wang, Zhihao Hao, Jingjing Chen, Nanping Wu

**Affiliations:** ^1^ Cell Biology Research Platform, Jinan Microecological Biomedicine Shandong Laboratory, Jinan, Shandong, China; ^2^ State Key Laboratory for Diagnosis and Treatment of Infectious Diseases, National Clinical Research Center for Infectious Diseases, National Medical Center for Infectious Diseases, Collaborative Innovation Center for Diagnosis and Treatment of Infectious Diseases, The First Affiliated Hospital, Zhejiang University School of Medicine, Hangzhou, Zhejiang, China; ^3^ Clinical Laboratory, Qingdao Hospital, University of Health and Rehabilitation Sciences (Qingdao Municipal Hospital), Qingdao, China; ^4^ Department of Clinical Laboratory, Shandong Provincial Third Hospital, Shandong University, Jinan, Shandong, China; ^5^ School of Public Administration, Guangxi University, Nanning, Guangxi, China

**Keywords:** human immunodeficiency virus and tuberculosis, disability-adjusted life years, age-standardized rate, disease burden, Bayesian age-period-cohort

## Abstract

**Objective:**

To assess sex, age, regional differences, and the changing trend in human immunodeficiency virus and tuberculosis (HIV-TB) in different regions from 1990 to 2021, and project future trends.

**Methods:**

Global Burden of Disease Study 2021 data were analyzed to assess HIV-TB incidence, death, prevalence, and DALY rates from 1990 to 2021, including different types of TB co-infections (drug-susceptible, multidrug-resistant, and extensively drug-resistant). Bayesian age-period-cohort models were used to forecast age-standardized DALY rates through 2035.

**Results:**

In 2021, there were approximately 1.76 million HIV-TB infections and 200,895 deaths globally. The highest burden of HIV-DS-TB and HIV-MDR-TB was found in Southern Sub-Saharan Africa, while HIV-XDR-TB was most prevalent in Eastern Europe. The co-infection burden was highest among individuals aged 30–49. Key risk factors were unsafe sex, drug use, and intimate partner violence, with regional variations. The global burden of HIV-TB remains high, and age-standardized DALY rates are expected to increase in the coming years, especially in regions with low socio-demographic indices (SDI).

**Conclusion:**

The burden of HIV-TB co-infection correlates with the socio-demographic index (SDI): countries with a low SDI have a higher burden. Therefore, clinical diagnosis and treatment in such areas are more challenging and may warrant more attention. High death rates underscore the importance of early management.

## 1 Introduction

Tuberculosis (TB) is a significant contributor to the global disease burden, causing more than one million deaths yearly (1). Unfortunately, 90%–95% of the TB burden is concentrated in low-income and middle-income countries (2). About one-third of deaths among people living with HIV are attributed to TB (3). Human immunodeficiency virus (HIV) and TB are two of humanity's most challenging infectious diseases, placing a considerable burden on healthcare systems worldwide. Over time, HIV and TB have influenced each other's natural history and pathogenesis, and their co-infection between has led to significantly higher incidence and death (3). Recently, the burden of disease and death in HIV-TB co-infected populations has increased, with the risk of active TB being 19 times higher in HIV-infected individuals than in HIV-negative individuals (4). At the same time, HIV infection also further amplifies the local TB epidemic, and there is a positive correlation between HIV infection and multidrug-resistant TB (5, 6). Globally, there are more than 1.3 million cases of HIV-associated TB each year, resulting in nearly 500,000 deaths; sub-Saharan Africa accounts for an estimated 79% of the burden of this disease (7). In areas with high rates of HIV and TB, particularly in Africa and Asia, TB mortality also increased during the early stages of the HIV pandemic (8) Meanwhile, the short-term strategy that WHO has directly observed, which relies on a process of passive TB case detection, has helped control TB in many parts of the world, but has not worked in countries where HIV is endemic (with infection rates exceeding 1% of the general population) (7). Therefore, exploring the global changing trends of HIV-TB co-infection is of great significance for preventing the spread of epidemics.

Meanwhile, TB could accelerate progression by increasing HIV replication (9). Evidence suggested that HIV development was accelerated partly via the increased systemic immune activation by HIV-TB. In TB infection, several complex cellular mechanisms might contribute to accelerated viral replication (4). This has led to significantly higher mortality than in HIV only cases (10). Although progress has been made in reducing TB-related deaths among people living with HIV resulting from developments in diagnosis, treatment, and antiretroviral HIV therapy, multidrug-resistant TB (MDR-TB) is now emerging as a source of concern (11). Drug resistance leads to further spread of both pathogens, and people living with HIV and HIV-AIDS-extensively drug-resistant tuberculosis (HIV-XDR-TB) face fewer treatment options and poorer treatment outcomes (12). Thus, the burden of HIV-XDR-TB infection should be highlighted.

While TB and HIV are important global public health issues, available data on HIV-TB risk factors are limited. Such information is critical to provide a comprehensive view to inform policy and improve care and support for these populations (13). This study analyzes the latest data on the burden of HIV-TB, assessing the global, regional, and national epidemiological characteristics of HIV-TB, including HIV/AIDS-drug-susceptible tuberculosis (HIV-DS-TB), HIV/AIDS-multidrug-resistant tuberculosis without extensive drug resistance (HIV-MDR-TB), and HIV-XDR-TB. Risk factors were assessed to provide robust and detailed evidence for policy and planning decisions.

## 2 Material and methods

### 2.1 Study design

This study is a secondary analysis of the 2021 Global Burden of Disease (GBD) database. Data were extracted from vital registration systems, verbal autopsies, censuses, household surveys, disease-specific registries, health service contact data, and other sources (14). GBD 2021 provides an accurate and comprehensive summary of the global disease burden for 371 diseases and injuries for 1990–2021 by age and sex and systematically analyzes the burden of disease due to risk factors for comparison between different countries and regions (15).

### 2.2 Patient and public involvement

It was not appropriate or possible to involve patients or the public in the design, conduct, reporting or dissemination plans of our research.

### 2.3 Date sources

Data to estimate the burden of HIV-TB are available in the GBD 2021 (https://vizhub.healthdata.org/gbd-results/). The GBD 2021 provides comprehensive global disease information, such as incidence number/rate, prevalence number rate, death number/rate, years of life lost (YYLs), years lived with disability (YLDs), and disability-adjusted life years (DALYs) attributable to HIV-TB populations from 1990 to 2021, where DALYs = YYLs + YLDs (16). The 95% uncertainty intervals (UIs) presented were derived from the GBD 2021 dataset. 95% UIs were generated for all final estimates as the 2.5th and 97.5th percentiles values of 500 draws (14, 17). The burden of disease is estimated using DALYs (18), expressed as the sum of years of life lost due to disease, with one year of healthy life lost representing one DALY (19). Years of life are estimated based on cause, location, age group, sex, and year in the GBD 2021. In addition, studies in GBD 2021 include broader estimates of age, sex, location, and year. GBD world population standards were used to calculate the age-standardized rate (ASR). All rates are reported per 100,000 population.

The data for this study was extracted from the GBD 2021 database. Previous GBD studies have reported standardized methods for data screening, cleaning, and generation (14, 20). The GBD project inputs data from censuses, disease registries, vital statistics, civil registries, satellite surveillance, health service records and other sources. GBD's Bayesian meta-regression tool DisMod-MR 2.1 was used to check and adjust data for bias by cross-validation. The estimates in the GBD project are updated each year by adding new available data and using more appropriate methods (21).

The database provides an accurate and comprehensive summary of the global burden of disease for 371 diseases and injuries from 1990 to 2021, aggregated by age and sex. It systematically analyses the burden of disease caused by risk factors for comparison across countries and regions, representing the largest and most comprehensive global observational epidemiological survey database to date (14). A detailed description of the raw data and general methodology for the GBD 2021 study has been presented in previous studies (15).

### 2.4 Definition

The number of risk factors assessed in GBD 2021 varies by condition on the basis of evidence of association and available data (15). The GBD 2021 study reported on the relationship between HIV-TB and risk factors. Similar to etiology, GBD classifies risk factors into four classes. From the broadest (level 1) to the most specific (level 4). In addition to the particular risk factors described above, we also assessed behavioral risks. The definitions of risk factors, as well as risk groups and further details of risk factors, have been described previously (15, 22). The main risk factors included in this study were drug use, unsafe sex, and intimate partner violence.

The socio-demographic index (SDI) is a new indicator that comprehensively represents social and demographic development. In 2015, the indicator was proposed by the GBD. The SDI primarily comprises crucial factors, such as a country's per capita income, average educational attainment, and fertility rates, which influence population health levels. It is a composite measure of lag-distributed income per capita, average years of education for those aged 15 years or older, and fertility rates among females younger than 25 years (14). Using the SDI, researchers can further investigate the relationship between the disease burden and core socio-economic factors that promote population health and provide policymakers with rationalized recommendations (1).

### 2.5 Statistical analysis

Our study uses the Bayesian APC (BAPC) model for predictive analyses. The BAPC model can be viewed as a Bayesian extension of the traditional APC model, which introduces a Bayesian approach to the framework of the APC model and utilises Bayesian inference for estimating parameters (23). The Bayesian approach allows prior information to be introduced into the model, and the posterior distribution is obtained by calculating the prior distribution and the observed data, helping to estimate age, period, and cohort effects more accurately (24). The BAPC model is able to handle uncertainty better, its estimation method can provide more robust predictions when data are limited or have large fluctuations. In practice, BAPC models typically use Markov Chain Monte Carlo (MCMC) methods or Integrated Nested Laplace Approximation (INLA) to estimate posterior distributions (24, 25). The plausibility of the BAPC model has been demonstrated previously (26, 27).

The Bayesian Age-Period-Cohort (BAPC) model is particularly advantageous for this study because it effectively captures the effects of age, time, and cohort simultaneously. This approach surpasses traditional time series analyses by allowing us to deconstruct and quantify the contributions of these factors to the evolution of disease burden, leading to more comprehensive projections (28). HIV-TB burden can exhibit nonlinear changes over time, influenced by a myriad of social, economic, and policy factors. The BAPC model, utilizing a Bayesian framework, dynamically adjusts predictions to accommodate these complexities, thereby enhancing accuracy. This study used the model to predict HIV-TB age-standardized DALY rates and found that the burden of disease is higher in the coming years.These findings can increase awareness of HIV-TB. This is critical for informing policy decisions and optimizing resource allocation.

The data on the incidence, prevalence, deaths, and DALYs in the HIV-TB population were extracted for stratification and comparative analysis, including relevant indicators such as sex, age, region, and country, to explore the changing trend of the disease burden in the HIV-TB population from 1990 to 2021. Meanwhile, the correlation between the global burden of HIV-TB and socio-economic development status was analyzed. To examine the burden of disease across multiple populations or time points, we used age-standardized rate (ASR) as a composite indicator to analyze the age-specific burden associated with different years. ASR for the HIV-TB population outcomes was primarily based on data from the GBD 2021. This study extracted data related to global HIV-TB incidence, death, prevalence, and DALY rates for HIV-DS-TB, HIV-MDR-TB, and HIV-XDR-TB and analyzed the age data into 20 age groups (from <5 year to 95 + years).

We used R version 4.2.1 [R Core Team (2021). A Bayesian age-period-cohort (BAPC) analysis in R using the BAPC and INLA packages was performed. R: A language and environment for statistical computing. R Foundation for Statistical Computing, Vienna, Austria. https://www.R-project.org/.] for data analyses.

## 3 Results

### 3.1 The global burden of HIV-Tb co-infection

**Figure 1:**
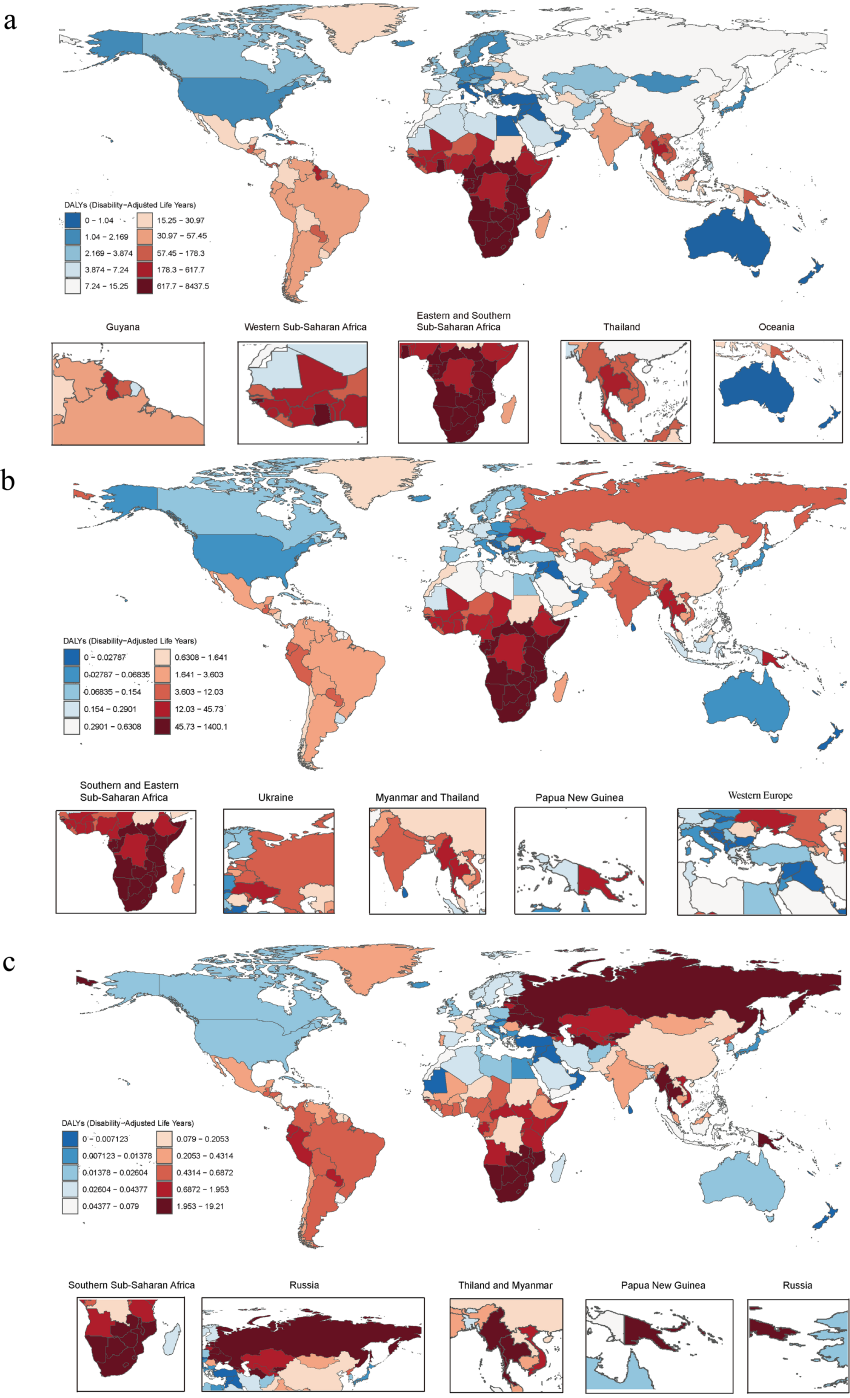
**(a)** Age-standardized DALY rates of HIV-DS-TB, **(b)** age-standardized DALY rates of HIV-MDR-TB, **(c)** age-standardized DALY rates of HIV-XDR-TB per 100,000 cases in 2021 by country. DALY, disability-adjusted life year; HIV-DS-TB, HIV/AIDS-drug-susceptible tuberculosis; HIV-MDR-TB, HIV/AIDS-multidrug-resistant tuberculosis without extensive drug resistance; HIV-XDR-TB, HIV-AIDS-extensively drug-resistant tuberculosis.

**Figure 2:**
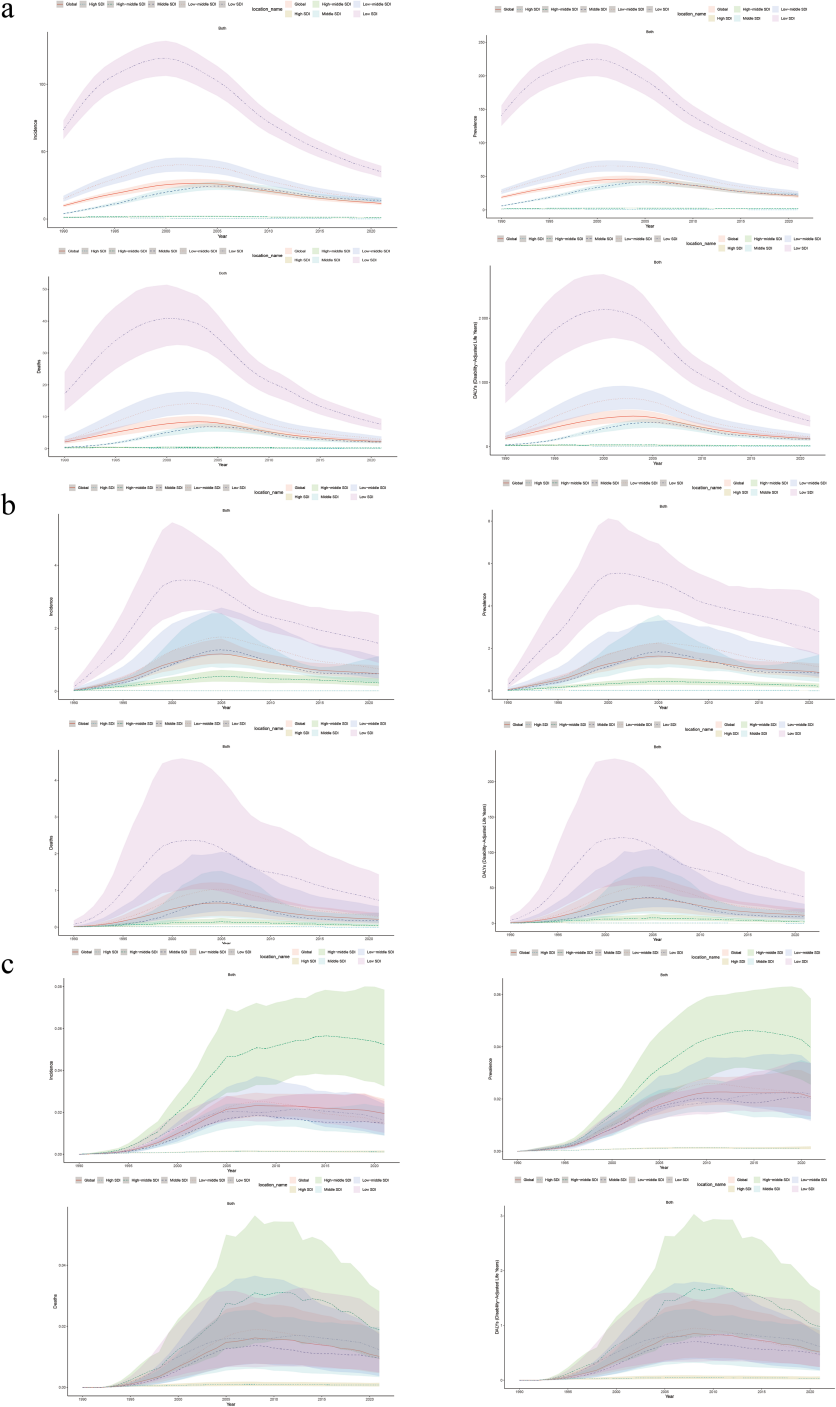
**(a)** Age-standardized incidence, prevalence, death, and DALY rate by global, high SDI, high-middle SDI, middle SDI, low-middle SDI, and low SDI in HIV-DS-TB from 1990 to 2021. **(b)** Age-standardized incidence, prevalence, death, and DALY rate by global, high SDI, high-middle SDI, middle SDI, low-middle SDI, and low SDI in HIV-MDR-TB from 1990 to 2021. **(c)** Age-standardized incidence, prevalence, deaths and DALY rate by global, high SDI, high-middle SDI, middle SDI, low-middle SDI, and low SDI in HIV-XDR-TB from 1990 to 2021. SDI, socio-demographic index.

In 2021, there were 1,682,114.84 (95% UI 1,494,990.34–1,881,081.77) prevalence cases, 182,597.43 (95% UI 1,141,923.37–225,076.20) death cases, 955,220.60 (95% UI 854,660–1,075,239.95) incidence cases, and 9,910,865.78 (95% UI 7,825,965.89–12,110,494.40) number of DALYs of HIV-DS-TB (Supplementary Table S1). From 1990 to 2021, the total percentage change in the age-standardized incidence rate was −0.31 (95%UI −0.04 to 0.19), the change in the age-standardized death rate was 0.04 (95% UI −0.22 to 0.47), the change in the age-standardized prevalence rate was 0.09 (95% UI 0.03–0.17), and the change in the age-standardized DALY rate was −0.04 (95% UI −0.26 to 0.30) (Supplementary Table S1). Notably, the UIs in these high-burden regions were relatively large, indicating potential data limitations and variability in reporting. There were considerable differences between regions and countries, with high rates of age-standardized DALYs focused on regions such as Sub-Saharan Africa, and countries such as Lesotho, Eswatini, and Mozambique (Figure 1a). High age-standardized incidence rates were concentrated in Southern Sub-Saharan Africa (Supplementary Figure S1a). High age-standardized death rate was concentrated in Eastern Sub-Saharan Africa (Supplementary Figure S1b), and high age-standardized prevalence rate was concentrated in Southern Sub-Saharan Africa (Supplementary Figure S1c). The global age-standardized DALY rate trended increased from 1990 to 2003 and declined from 2004 to 2021 (Figure 2a).

In 2021, for HIV-MDR-TB there were 71,455.41 (95% UI 48,998.78–106,009.42) prevalence cases, 17,457.58 (95% UI 7,574.38–32,229.49) deaths cases, 45,589.40 (95% UI 31,326.35–66,723.02) incidence cases, and the number of DALYs 925,471.28 (95% UI 413,529.64–1,668,293.09) (Supplementary Table S2). The total percentage change in the age-standardized incidence rate from 1990 to 2021 was 14.44 (95% UI 7.52–25.68), the total percentage change in the age-standardized rate from 1990 to 2021 for death was 14.58 (95% UI 8.31–27.16), the total percentage change in the age-standardized rate from 1990 to 2021 for prevalence was 15.93 (95% UI 8.37–28.21), and the total percentage change in the age-standardized DALY rate was 14.05 (95% UI 7.87–26.04) (Supplementary Table S2). High age-standardized DALY and incidence rates were concentrated in the Southern Sub-Saharan Africa and Eastern Sub-Saharan Africa (Figure 1b; Supplementary Figure S2a). High age-standardized death rates and age-standardized prevalence rate were mainly concentrated in the Southern Sub-Saharan Africa region (Supplementary Figures S2b and S2c). The global age-standardized DALY rates increased from 1990 to 2005 and decreased from 2005 to 2021 (Figure 2b).

In 2021, for HIV-XDR-TB, there were 1,726.75 (95% UI 1,241.46–2,426.98) prevalence cases, 840.00 (95% UI 385.44–1,491.72) deaths cases, 1,606.42 (95% UI 1,163.94–2,182.83) incidence cases, and the number of DALYs 42,094.84 (95% UI 19,698.03–74,093.39) (Supplementary Table S3). High age-standardized DALY rates were mainly concentrated in Eastern Europe and Southern Sub-Saharan Africa (Figure 1c). High age-standardized incidences were focused on Eastern Europe and Southern Sub-Saharan Africa (Supplementary Figure S3a). High age-standardized death rates were concentrated in the countries of Eastern Europe and Southern Sub-Saharan Africa (Supplementary Figure S3b). High age-standardized prevalence rates were concentrated in the countries of Eastern Europe and Southern Sub-Saharan Africa (Supplementary Figure S3c). From 1990 to 2008, the age-standardized DALY rate of HIV-XDR-TB showed an upward trend, but declined from 2009 to 2021 (Figure 2c); the downward trend in 2008–2021 was slower than that of HIV-DS-TB and HIV-MDR-TB (Figure 2a–c).

### 3.2 Global and SDI regions

Age-standardized rates varied considerably between regions, mainly in low and middle SDI. The increase in HIV-DS-TB and HIV-MDR-TB burden was associated with SDI. Low SDI areas had higher age-standardized incidence, death, prevalence, and DALY rates. Conversely, the age-standardized prevalence was lowest in areas with higher SDI levels (Figures 2a,b). In contrast, the incidence, death, prevalence, and DALY rate of age-standardized in HIV-XDR-TB was significantly higher in high-middle SDI than in other regions (Figure 2c).

In 2021, in HIV-DS-TB, the total percentage change in age-standardized incidence rate, death rate, prevalence rate, and DALY rate from 1990 to 2021 in low SDI areas were −0.50 (95% UI −0.54 to 0.46), −0.57 (95% UI −0.69 to 0.37), −0.54 (95% UI −0.57 to 0.50), and −0.61 (95% UI −0.71 to 0.45), respectively (Supplementary Table S1). In HIV-MDR-TB, the total percentage change in age-standardized incidence, death, prevalence, and DALY rates in low SDI areas were 9.35 (95% UI 3.27 to -22.32), 8.62 (95% UI 3.10 to −25.27), 8.99 (95% UI 3.09 to −21.03), and 7.86 (95% UI 2.74–22.12), respectively (Supplementary Table S2). In HIV-XDR-TB, the tatal percentage change in age-standardized death and DALY rates were higher in high-middle SDI areas than in other areas, at 39.19 (95% UI 13.89–110.54) and 35.64 (95% UI 13.01–97.71), respectively (Supplementary Table S3).

In conclusion, in HIV-DS-TB and HIV-MDR-TB, low SDI regions had significantly higher disease burden rates than high SDI, high-middle SDI, middle SDI, and low-middle SDI. While in HIV-XDR-TB, high-middle SDI regions had higher disease burden rates than the other SDI areas.

### 3.3 Distribution of 21 disease burden regions in 2021

In 2021, Southern Sub-Saharan Africa had the highest rate of age-standardized DALY rates, followed by Eastern Sub-Saharan Africa (Supplementary Table S4). The age-standardized DALY rate varied widely across regions, ranging from 0.62 (95% UI 0.38–0.97) in Australasia to 3,400.80 (95% UI 2,869.16–3,753.34) in Southern Sub-Saharan Africa (Supplementary Table S4).

**Figure 3:**
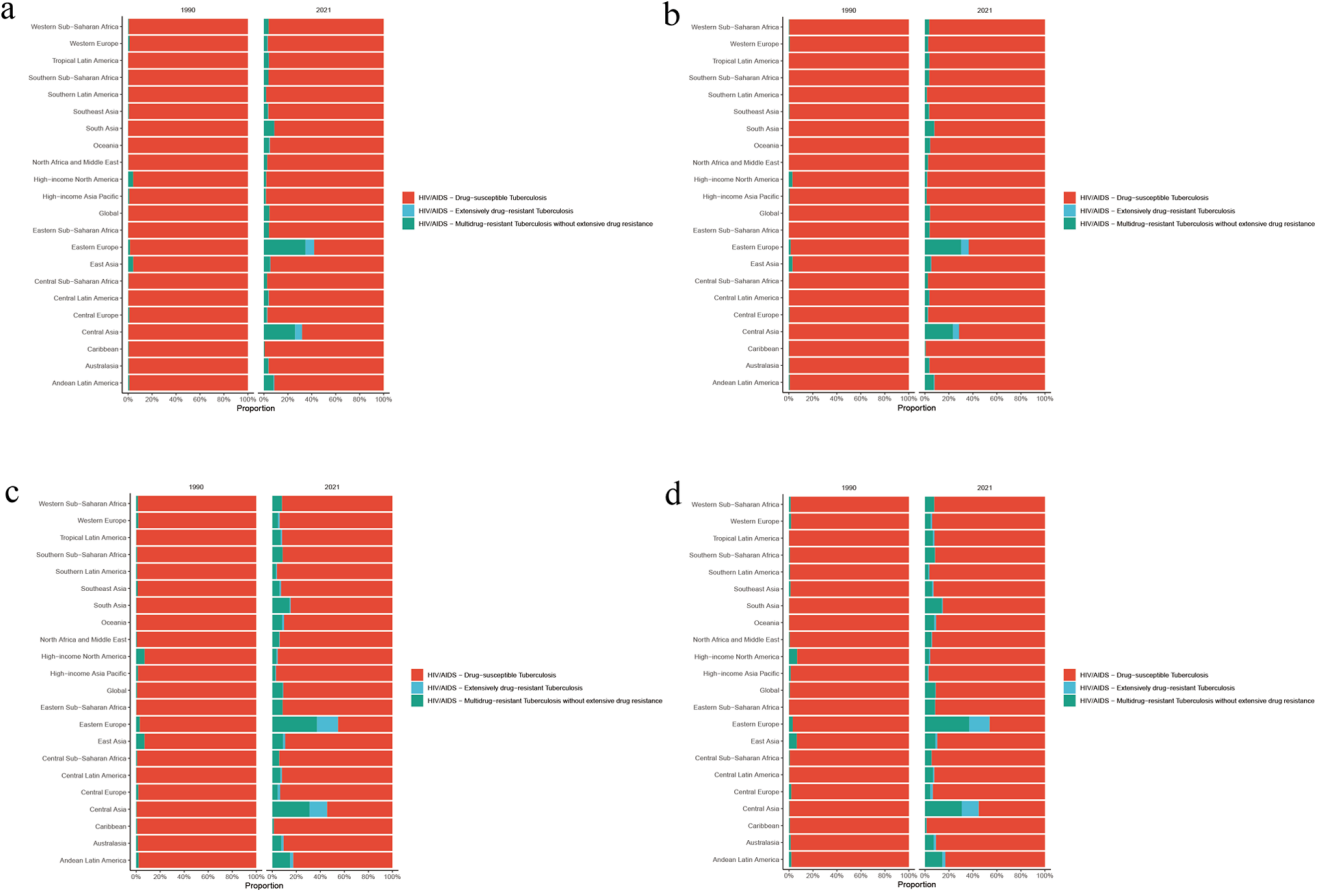
**(a)** Age-standardized incidence rate, **(b)** age-standardized prevalence rate, **(c)** age-standardized deaths rate, and **(d)** age-standardized DALY rate of HIV-DS-TB, HIV-MDR-TB, and HIV-XDR-TB in 21 disease burden regions in 2021.

HIV-MDR-TB had the highest burden in Southern Sub-Saharan Africa, followed by Eastern Sub-Saharan Africa (Supplementary Table S5). In 2021, the number of incidences, death, prevalence and DALYs were 131,23.40 (95% UI 6,235.44–28,458.34), 4,876.84 (95% UI 1,803.52–10,740.13), 20,032.52 (95% UI 9,192.34–42,752.54) and 250,958.97 (95% UI 94,722.13–543,970.49) in Southern Sub-Saharan Africa (Figure 3a–d; Supplementary Table S2). The age-standardized rates of incidence, death, prevalence and DALY were 16.40 (95% UI 7.79–35.65), 6.13 (95% UI 2.27–13.47), 25.07 (95% UI 11.59–53.46), and 304.42 (95% UI 114.45–658.52), respectively (Figure 3a–d; Supplementary Table S5). The number of incidences, death, prevalence and DALYs were 12,361.93 (95% UI 6,912.27–20,663.83), 5,925.99 (95% UI 2,418.74–12,025.23), 21,594.97 (95% UI 12,290.69–36,375.37), and 331,047.86 (95% UI 136,235.45–659,836.98) in Eastern Sub-Saharan Africa (Figure 3a–d; Supplementary Table S2). Age-standardized rates of incidence, death, prevalence and DALY were 3.82 (95% UI 2.16–6.26), 1.81 (95% UI 0.74–3.72), 6.70 (95% UI 3.88–11.16) and 90.59 (95% UI 37.58–182.97), respectively (Figure 3a–d; Supplementary Table S5).

HIV-XDR-TB had the highest Age-standardized DALY rates in Eastern Europe, followed by Southern Sub-Saharan Africa (Figure 3d; Supplementary Table S6). In addition, the results showed that HIV-XDR-TB was a significant threat to Oceania, Eastern Sub-Saharan Africa, and Central Asia (Figure 3d).

### 3.4 Sex and age

**Figure 4:**
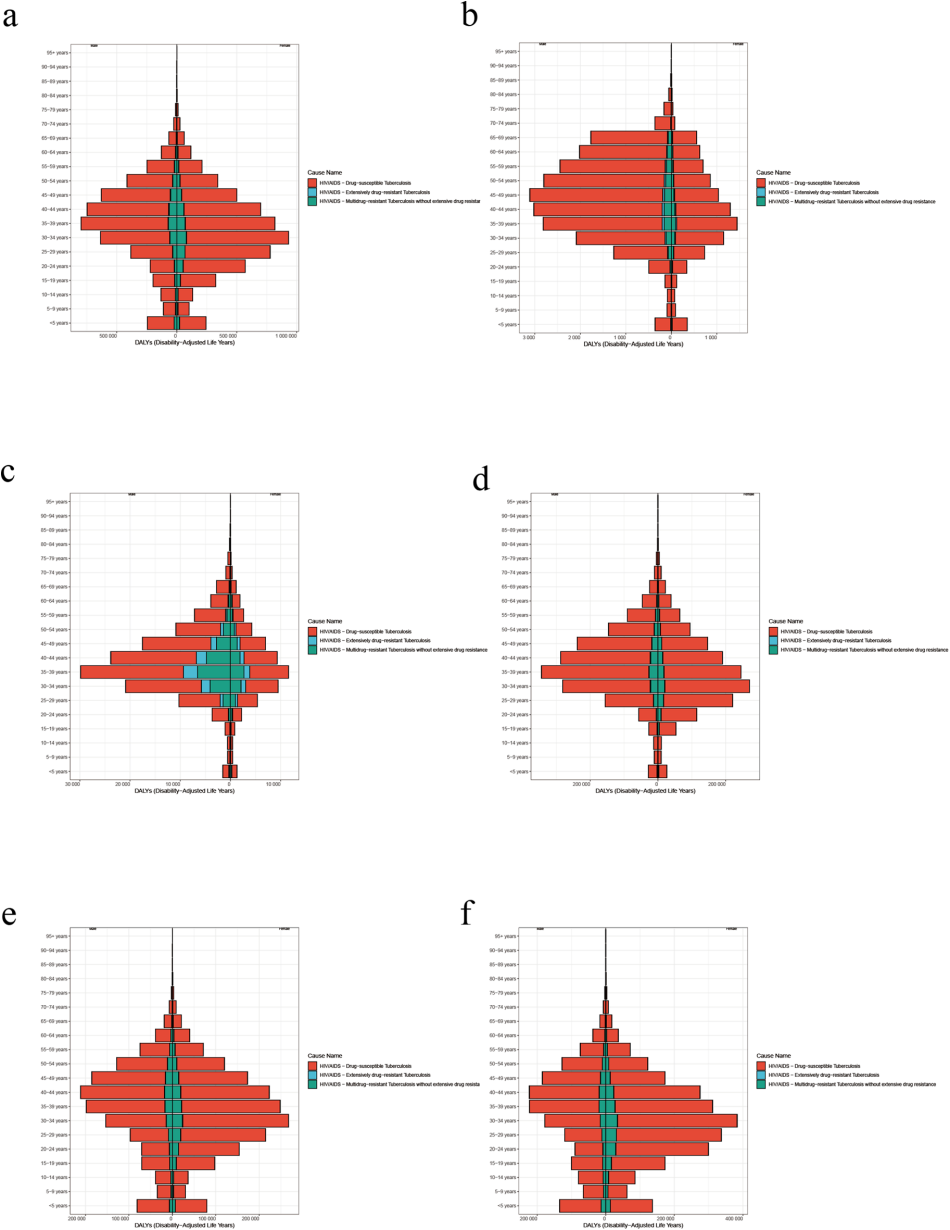
**(a)** Female and male age-standardized DALY rate by age in global in 2021. **(b)** Female and male age-standardized DALY rate by age in high SDI in 2021. **(c)** Female and male age-standardized DALY rate by age in high-middle SDI in 2021. **(d)** Female and male age-standardized DALY rate by age in middle SDI in 2021. **(e)** Female and male age-standardized DALY rate by age in Low-middle SDI in 2021. **(f)** Female and male age-standardized DALY rate by age in low SDI in 2021.

The burden of the disease varied between males and females in the same age group in different regions (Figure 4). High rates of age-standardized DALYs in females with HIV-DS-TB were concentrated in the 30–39 year age group, while cases in males were focused in the 40–49 year age group (Figure 4).

Among females and male with HIV-MDR-TB, similar to HIV-DS-TB, high rates of age-standardized DALY were concentrated in the 35–39 year age group (Figure 4c). It is more significant in high-middle SDI area, and males are higher than females (Figure 4c).

In females with HIV-MDR-TB, high rates of age-standardized DALY rate were concentrated in the 30–39 year age groups, while high rates of age-standardized DALY in males were concentrated in the 35–39 year age group (Figure 4c). Globally, high SDI, high-Middle SDI, and middle SDI are represented by more males than females (Figures 4a–d) and Low-middle SDI and low SDI regions are represented by more females than males (Figures 4e,f).

### 3.5 Risk factors

**Figure 5:**
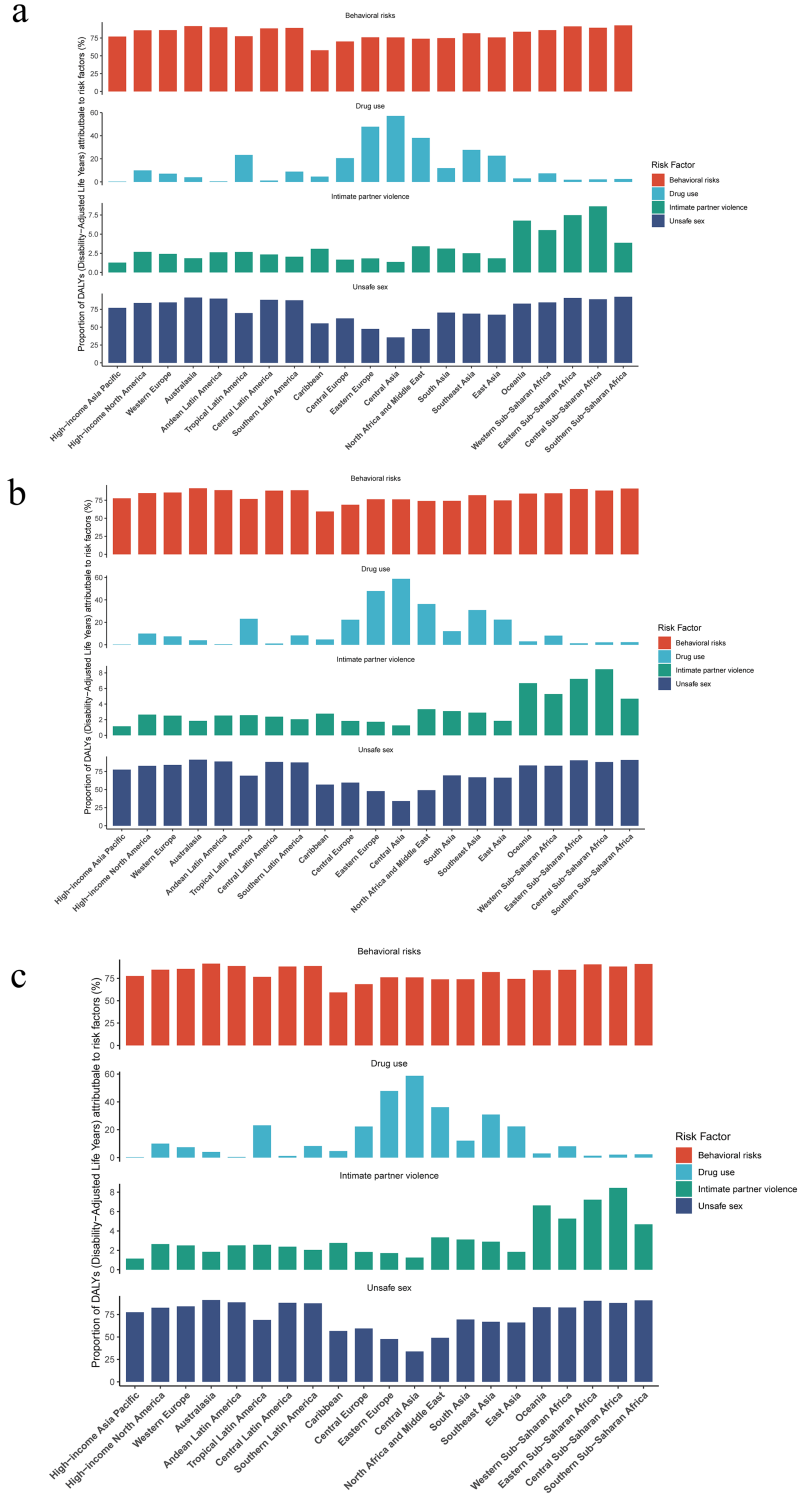
Risk factor analysis for **(a)** HIV-DS-TB, **(b)** HIV-MDR-TB and **(c)** HIV-XDR-TB in global and SDI regions in 2021.

In 2021, total HIV-TB was attributable to the for risk factors, including behavioral risks, drug use, intimate partner violence, and unsafe sex. The study showed that behavioral risks and unsafe sex were the main risk factor in the burden of HIV-DS-TB (Figure 5a). The top three regions for HIV-DS-TB due to unsafe sex are Southern sub-Saharan Africa, central sub-Saharan Africa, and Eastern sub-Saharan Africa (Figure 5a). The top three regions for HIV-DS-TB due to intimate partner violence are oceania, central sub-Saharan Africa, and Eastern sub-Saharan Africa (Figure 5a). The top three regions for HIV-DS-TB due to drug use are Central Asia, Eastern Europe, and North Africa and Middle East (Figure 5a).

In HIV-MDR-TB, the top three regions for the impact of unsafe sex were Southern sub-Saharan Africa, central sub-Saharan Africa, and Eastern sub-Saharan Africa (Figure 5b). The top three regions for the impact of intimate partner violence were Central Sub-Saharan Africa, Eastern Sub-Saharan Africa, and Oceania (Figure 5b). The top three regions for the impact of drug use were central Asia, Eastern Europe, and North Africa and Middle East (Figure 5b).

The study showed that drug use and unsafe sex significantly influenced the burden of HIV-XDR-TB (Figure 5c). The top three regions for the impact of drug use were central Asia, Eastern Europe, and North Africa and Middle East (Figure 5c). The top three regions for the effects of intimate partner violence were Central Sub-Saharan Africa, Eastern Sub-Saharan Africa, and Oceania (Figure 5c). The top three regions for the impact of unsafe sex were Southern Sub-Saharan Africa, Australasia, and Eastern Sub-Saharan Africa (Figure 5c).

### 3.6 Bayesian age–period–cohort analysis

**Figure 6:**
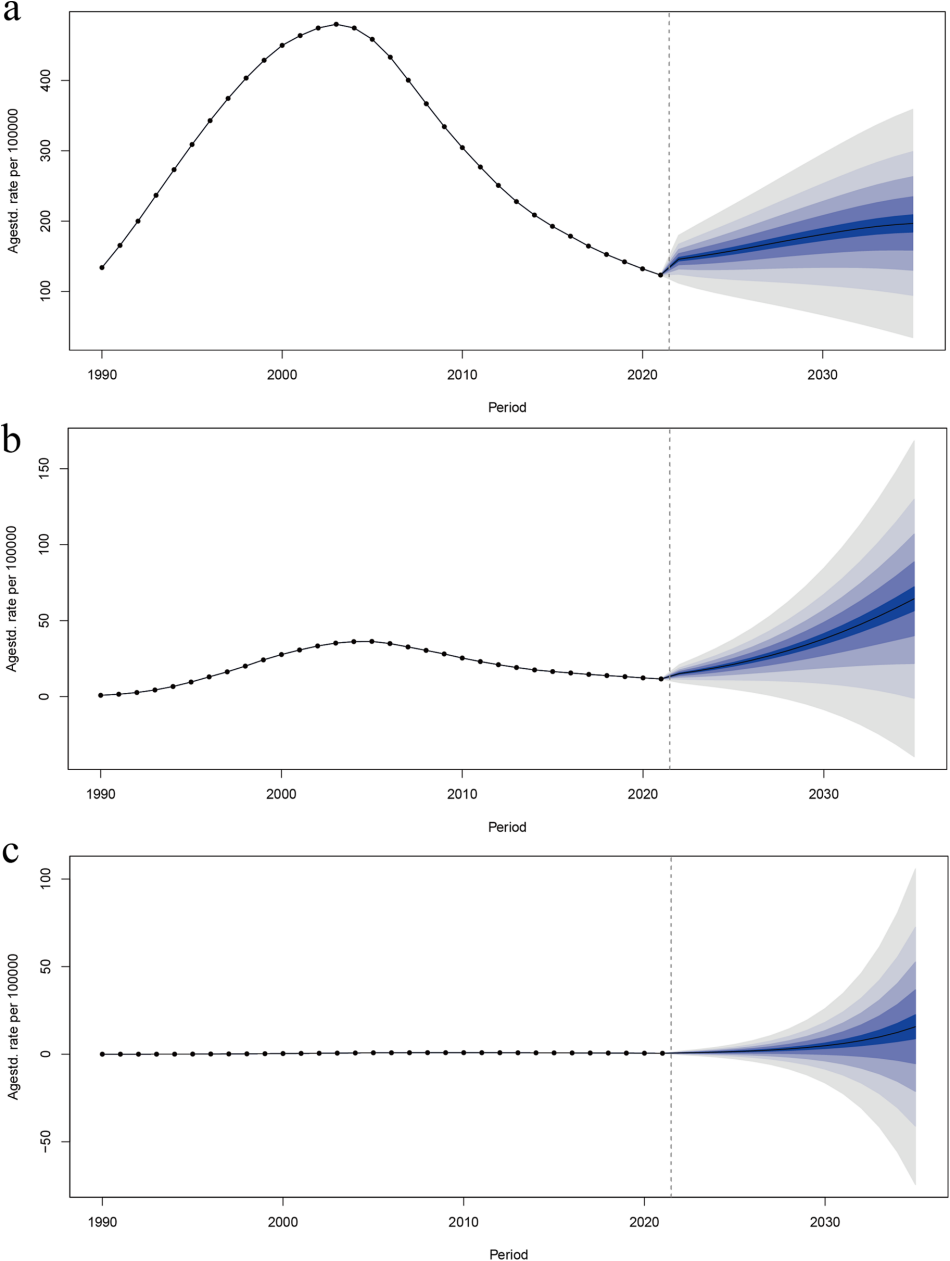
Trends in age-standardized DALY rates from 2021 to 2035 for **(a)** HIV-DS-TB, **(b)** HIV-MDR-TB, and **(c)** HIV-XDR-TB predicted by Bayesian age–period–cohort (BAPC) models.

Age-standardized DALY rates were projected to 2035 using the BAPC model. The results are shown in Figure 6, where age-standardized DALYs were predicted to increase in the HIV-DS-TB population, in the HIV-XDR-TB population, and in the HIV-MDR-TB population, and were shown to be higher age-standardized DALY rate occurs in the HIV-DS-TB population, with the fastest growing trend in the HIV-MDR-TB population (Figure 6).

HIV-DS-TB displayed an increasing trend from 1990 to 2003, followed by a decrease until 2021, and a slight uptick afterward (Figure 6a). Similarly, HIV-MDR-TB showed an upward trend from 1990 to 2005, then declined until 2021, before beginning to rise again (Figure 6b). In contrast, Figure 6c illustrates that HIV-XDR-TB has experienced a gradual increase from 1990 and is projected to continue this slow upward trend through 2030 (Figure 6c).

## 4 Discussion

HIV-TB is an emerging threat to global public health, and TB is the leading cause of death among people living with HIV globally (29). The global burden of HIV-TB has been studied by previous researchers (12), these studies mainly focused on exploring the incidence of HIV-TB in 204 countries and regions from 1990 to 2021. This study complements previous research and uses the newly released GBD 2021 to assess global HIV-TB trends, which were not reported in previous studies.

The current study revealed that HIV-TB increased markedly from 1990 to 2021 and that HIV-TB remains an unresolved global issue (30). In 2021, HIV-TB caused 1,755,296.99 (95% UI 1,545,230.58–1,989,518.17) HIV-TB infections globally and 200,895.02 (95% UI 149,883.19–258,797.41) deaths cases among people living with HIV. Despite the expansion of HIV-TB services, globally, the risk of HIV-TB remains high. Studies have shown that interventions that increase the likelihood of early diagnosis of HIV and TB can reduce mortality in adults with HIV-TB co-infection (30).

From 1990 to 2021, HIV-MDR-TB and HIV-XDR-TB showed an increasing and then decreasing trend, likely because of the worldwide expansion of antiretroviral therapy. After 2004, a WHO policy document on collaborative action was published on HIV-TB and significant progress in HIV-TB control has been achieved (31, 32). Nevertheless, the increasing trend of HIV-TB could not be reversed for a number of reasons, such as inadequate treatment of MDR-TB and poor drug adherence. HIV-XDR-TB showed a non-significant decreasing trend globally and in different SDI regions, but also increased in high SDI regions after 2014, indicating that growing numbers of countries in high SDI regions are exposed to the threat of HIV-XDR-TB. Therefore, we should focus more on HIV-XDR-TB to effectively stop its global growth. For HIVXDR-TB, treatment adherence can be improved by optimising the treatment regimen, introducing more effective combinations of anti-tuberculosis drugs, shortening the treatment period and reducing side effects (33, 34). At the same time, a strict drug management system is in place to prevent the unregulated use of drugs (35). Timely detection and reporting of HIV-XDR-TB cases. In addition, countries should also collaborate on strategies to combat drug-resistant tuberculosis and share experiences in governance.

Our findings showed that the burden of HIV and TB co-infection was concentrated in Sub-Saharan Africa. Previous studies have shown that TB patients tested for HIV varied considerably, particularly among vulnerable groups at risk of HIV and TB simultaneously (36). The main reasons were poverty, economic hardship, and lack of medical resources. In addition, the high economic costs in low-income and middle-income countries, poor socio-economic conditions, and potentially modifiable risk factors, such as smoking, alcohol consumption, and diabetes, in the context of a high HIV epidemic, have contributed to the increased disease burden (2). Meanwhile, studies have shown that HIV-MDR-TB and HIV-XDR-TB have expanded into Oceania and Eastern Europe. Successful control requires improved diagnostic tests and a shorter preventive treatment duration, especially for HIV-XDR-TB (37).

In addition, based on the characteristics of this study that the HIV-TB burden is highly concentrated in low SDI areas, it is recommended that policymakers give priority support to low-income and high-burden areas when allocating resources. Consideration can be given to increasing diagnostic, treatment and monitoring resources in these areas through international aid and national public health budgets. For example, in areas with a high HIV-TB burden, such as sub-Saharan Africa, the supply of multidrug-resistant TB (MDR-TB) and extensively drug-resistant TB (XDR-TB) drugs should be increased to ensure that patients can receive timely and appropriate treatment. Finally, more stringent drug resistance management strategies are needed for areas and key populations with a high burden of MDR-TB and XDR-TB (38).

From 1990 to 2021, among females, the 30–34 age group had the highest prevalence of HIV-DS-TB and HIV-MDR-TB, and in males, the 35–39 age group had the high age-standardized DALY rate in global. In addition, among cases of HIV-XDR-TB, the burden of HIV-TB co-infection was higher among males than females in global, possibly because males exhibit lower levels of health care use because of fear or avoidance of diagnosis, and exhibit poorer adherence to treatment under social pressure (1). In addition, certain behavioral risk factors, such as higher rates of smoking, alcohol consumption, or substance abuse, are often more prevalent among men, which may lead to greater susceptibility to HIV-TB co-infection and other health complications. For example, men may be more exposed to environments that increase the risk of HIV and TB transmission, particularly in high-SDI areas where occupational and lifestyle factors play a role (1, 39).

In low-middle SDI and low SDI regions, the burden of HIV and TB co-infection is higher among women than men, probably because HIV disproportionately affects these countries and HIV is the strongest risk factor for progression from latent to active TB (1). In some economically backward regions, such as sub-Saharan Africa, women have a lower socioeconomic status and are more likely to be uneducated, unemployed and poor than men. Unequal power relations and women's subordinate status relative to men put women at a higher risk of HIV infection (40). And in high-SDI areas, women may interact more frequently with the health care system (e.g., for reproductive health), leading to earlier detection and management of HIV-TB, potentially affecting disability-adjusted life years (DALYs) at an earlier age range (1).

Given that high-burden areas are concentrated in low-income countries, targeted interventions for key populations, such as women and those aged 30–49, are essential. Strengthening health education, protection policies and health services for women, promoting community health initiatives and increasing the frequency of HIV and tuberculosis screening are critical to slowing the progression of the disease and reducing infectiousness (41). For women aged 30–34 and men aged 35–39, tailored prevention education is essential. Increasing healthcare utilization among men and addressing behavioural risks such as smoking and substance abuse can reduce co-infection rates. Empowering women in low SDI areas through education and socio-economic support further reduces their vulnerability, while integrating HIV and tuberculosis services can optimise resource allocation and intervention effectiveness (42).

Current study also found that for HIV-DS-TB, HIV-MDR-TB, and HIV-XDR-TB, the burden is higher in infants younger than five year. Although much progress has been made globally in addressing the TB and HIV/AIDS epidemic over the past 20 years, HIV and TB co-infection in children, particularly in the infant population, remains a significant challenge (43). Sub-Saharan Africa was the most severely affected region, with Southern Africa accounting for 22 of the 30 high-burden HIV-TB countries reported by the WHO. The reasons for this might be that the low education level of pregnant and breastfeeding women and the persistently high number of new infections among women of reproductive age over the past decade have led to a prominent problem of vertical mother-to-child transmission in some underdeveloped regions of Africa, such as Sub-Saharan Africa (44). Coupled with relatively scarce medical resources, children have received antiretroviral treatment at a lower level than adults in these regions. This study found that infants and young children under 5 years of age were particularly affected, highlighting the need for HIV TB planning for infants and young children. Strategies can be developed to focus on early detection and treatment of HIV TB in children to ensure timely and appropriate medical care (45). At the same time, maternal education and care should be strengthened, PMTCT programmes should be reinforced, women should be educated on HIV and TB prevention, safe breastfeeding, and the importance of antenatal care, and comprehensive PMTCT services should be implemented, including routine HIV testing of pregnant women and provision of antiretroviral therapy to reduce infant infections (45). In addition, as children receive lower levels of antiretroviral therapy than adult strategies, focus should be placed on increasing the availability and accessibility of paediatric antiretroviral agents to ensure that children receive the same level of care as adults (42). The findings of this study can inform policy makers of the urgent need to address HIV-TB co-infection in children. Policies can be developed to integrate HIV and TB services for children to ensure a more comprehensive and effective management approach to enhanced prevention, diagnosis, and treatment of HIV (43).

Economically driven populations are aging in some countries, and most older people lack knowledge about HIV prevention and high-risk sexual behavior (46). Coupled with the continued increase in life expectancy among people living with HIV under widespread coverage of antiretroviral therapy, the chances of TB co-infection are also likely to increase (12). In previous studies, the burden of AIDS in older age groups has been described, and the aging of people living with HIV has become a widespread phenomenon in current society (47). The impact of HIV-TB on older age groups needs to be considered. Therefore, the government needs to focus more on the impact of the disease burden in older patients with HIV-TB (12). To enhance HIV-TB management strategies, it is crucial to implement targeted education and awareness campaigns for older adults, integrate routine HIV and TB screening, customize treatment and care plans to address age-specific needs, train healthcare providers accordingly, and develop policies that allocate resources to meet the unique challenges faced by older adults with HIV-TB (47, 48).

The present study showed that the main risk factors for HIV and TB co-infection included drug use, unsafe sex, and intimate partner violence. Other risk factors associated with HIV-TB death have previously been reported to include low CD4 count, advanced HIV status, not receiving antiretroviral therapy, not receiving cotrimoxazole prophylaxis, older age, incarceration, low weight, and bed rest; and socio-economic factors such as low knowledge and socio-economic status, poor living conditions, and limited access to health care (49). These factors are further compounded in patients with HIV-TB. Thus, adequate social support, early detection, appropriate treatment, adequate access to health care, and optimal HIV-TB care integration and intervention are critical to addressing HIV and TB co-infection in these populations (30, 50).

Understanding trends and changes in HIV-TB can inform health policy and resource allocation (51). However, there are some limitations to this study. First, Although the study utilized the newly released GBD 2021 data, the accuracy of these data can still be limited by reporting discrepancies and potential underreporting in various regions, particularly in low-income areas with less robust health infrastructure such as Sub-Saharan Africa. Second, real data for 2022 and 2023 have not yet been released, so real data cannot be used to assess the model's forecast accuracy. Third, The GBD 2021 data used in this study came from a variety of secondary sources, and the quality and completeness of data reporting varies across countries and regions, with potential for bias. For example, HIV and TB data collection systems in low-income countries are relatively imperfect, which may lead to underestimation or overestimation of the burden of disease. Such bias may affect interregional comparisons of burden, especially in regions where data quality varies considerably.

## 5 Conclusion

HIV-TB is a global health concern, particularly in Sub-Saharan Africa. Recently, HIV-XDR-TB is concentrated in Oceania, Eastern Europe, and Asia, and the burden on children as well as older age groups should be given priority. HIV is the most significant risk factor for TB progression, and active prevention of HIV transmission effectively reduces the burden of HIV and TB co-infection. Moreover, strengthening surveillance, focusing on key risk factors, and targeting interventions to different groups in different regions are essential to control HIV-TB. Our findings will contribute to further epidemiological studies on HIV-TB worldwide.

## Data Availability

The original contributions presented in the study are included in the article/Supplementary Material, further inquiries can be directed to the corresponding author.
