# Supplementary material for: Global, regional, and national burden of HIV and tuberculosis and predictions by Bayesian age-period-cohort analysis: a systematic analysis for the global burden of disease study 2021
Source: Front Reprod Health. 2024 Dec 10;6:1475498. doi: 10.3389/frph.2024.1475498 (PMC11666487; doi:10.3389/frph.2024.1475498)
Supplement: Supplementary file 1 [file Table1.docx]

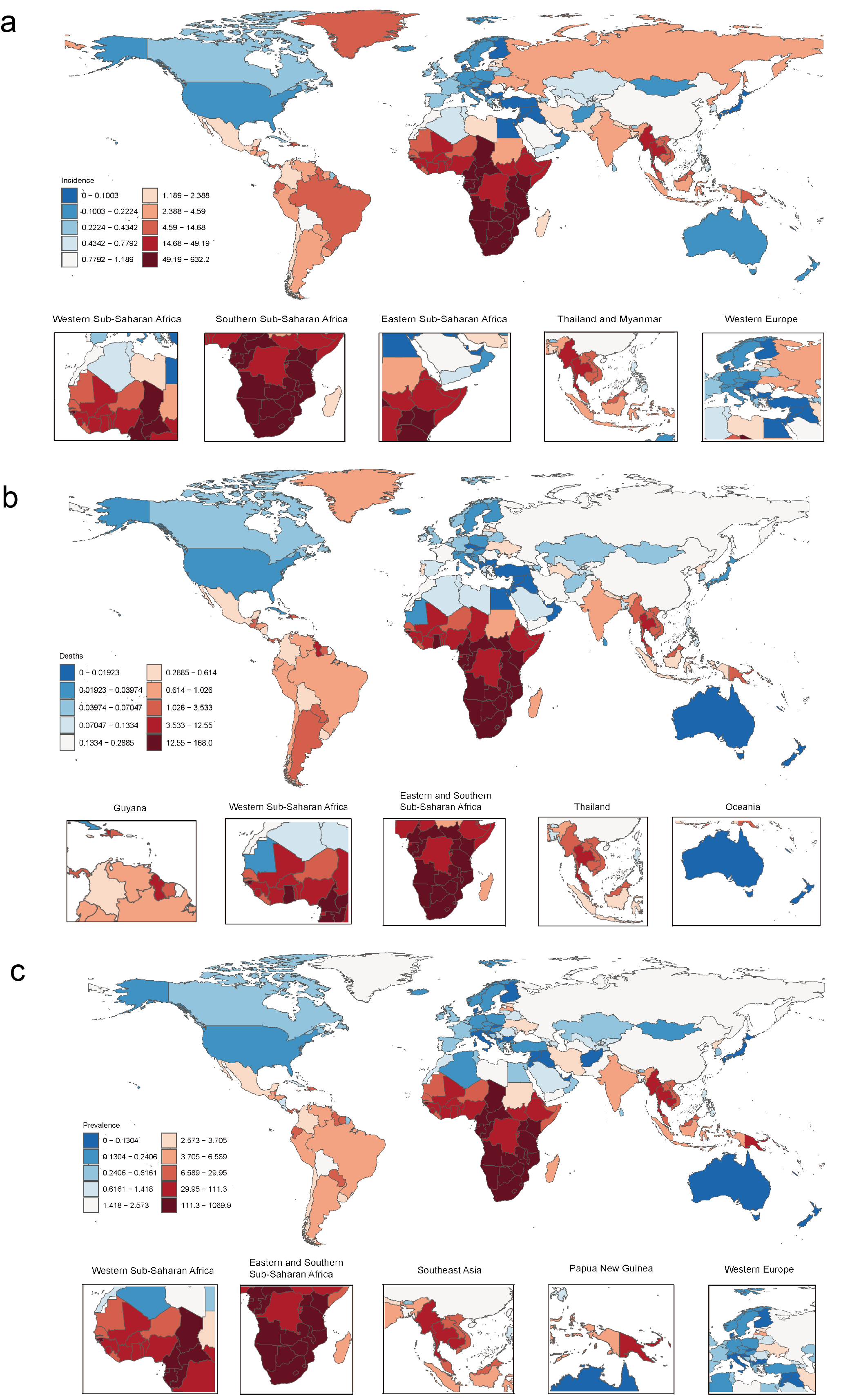


**Figure S1** (a) Age-standardized incidence rate, (b) age-standardized death rate, and (c) age‑standardized prevalence rate of HIV-DS-TB per 100,000 cases in 2021 by country. HIV-DS-TB, HIV/AIDS-drug-susceptible tuberculosis.


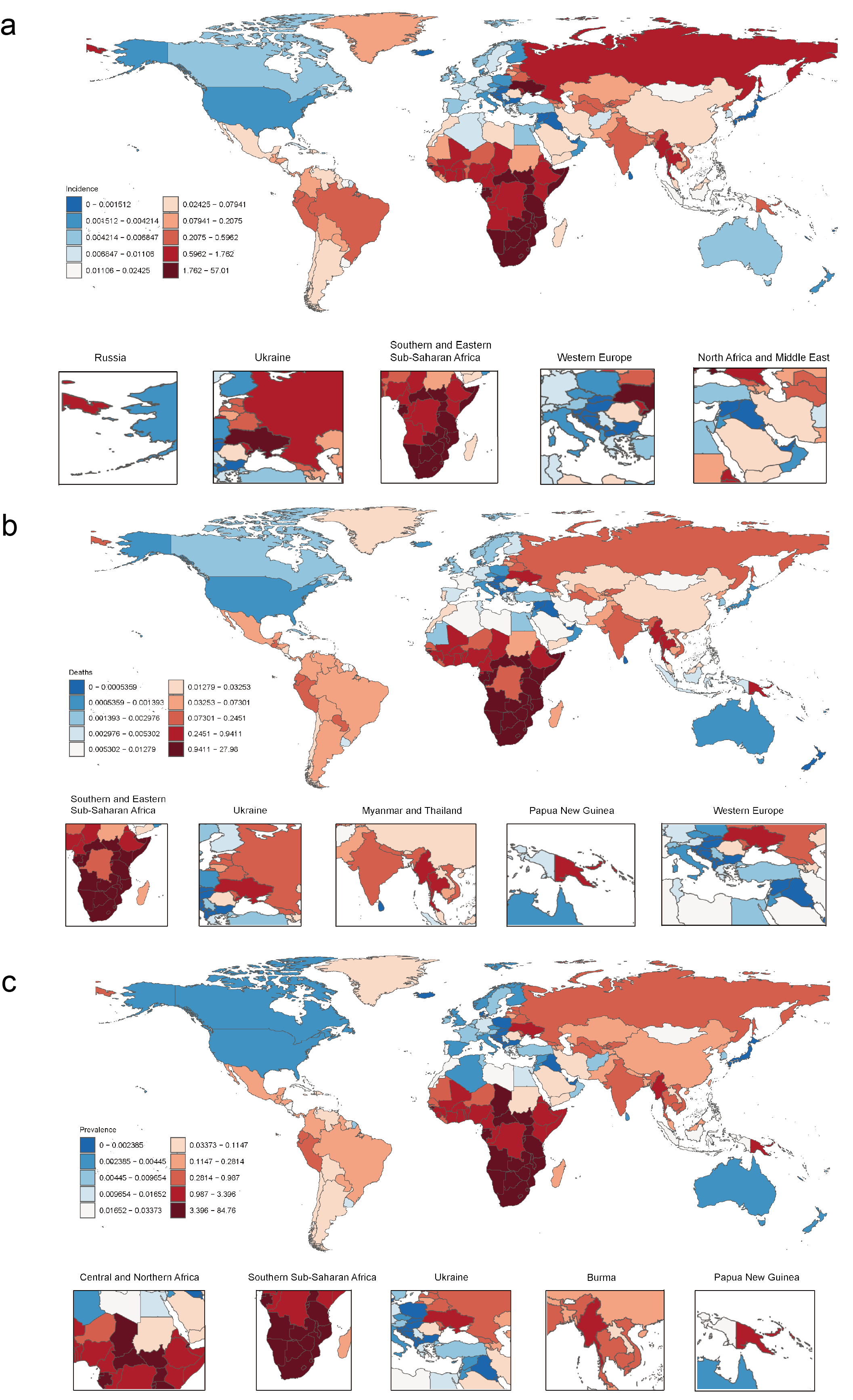


**Figure S2** (a) Age-standardized incidence rate, (b) age-standardized death rate, and (c) age‑standardized prevalence rate of HIV-MDR-TB per 100,000 cases in 2021 by country. HIV-MDR-TB, HIV/AIDS-multidrug-resistant tuberculosis without extensive drug resistance.


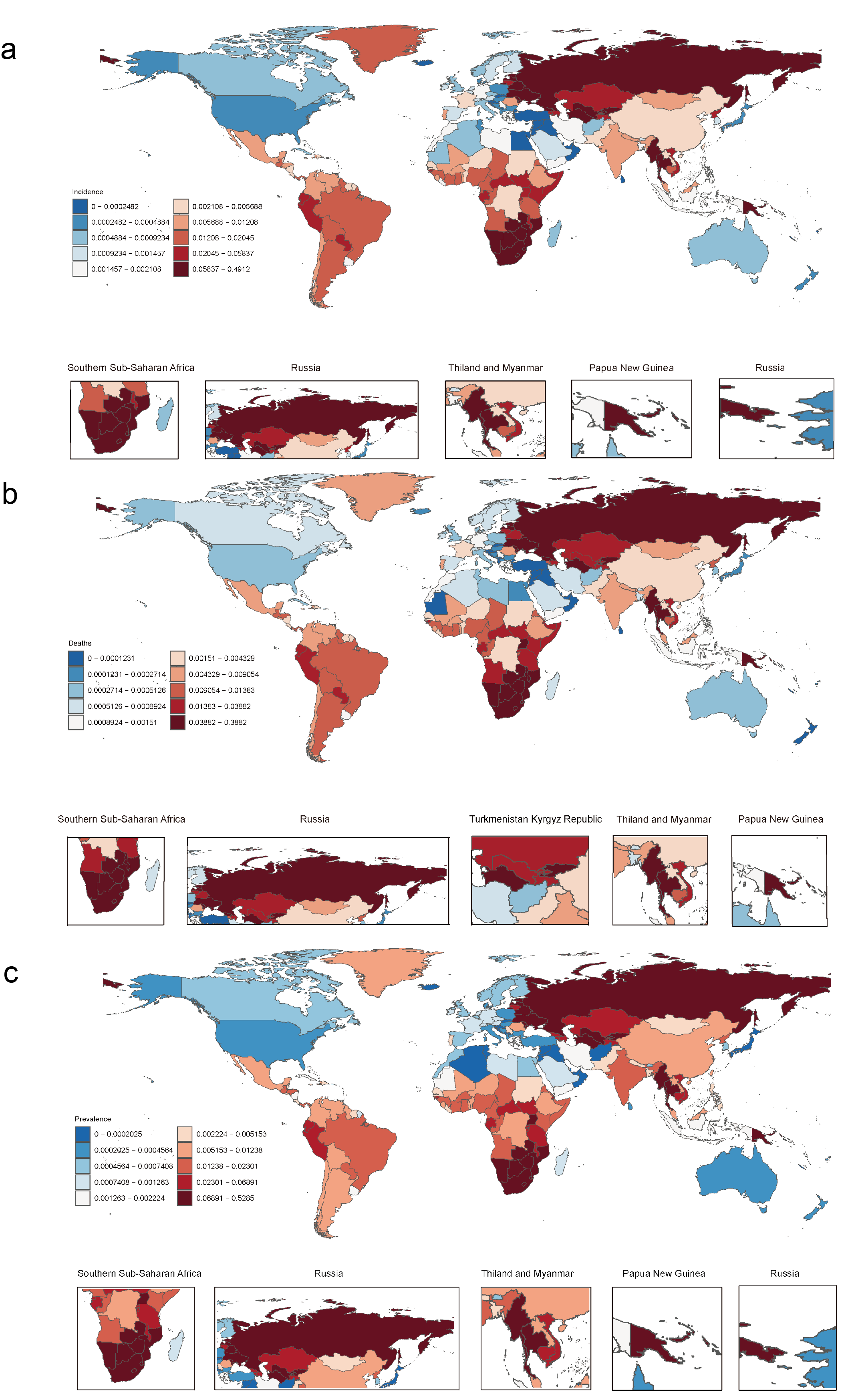


**Figure S3** (a) Age-standardized incidence rate, (b) age-standardized death rate, and (c) age‑standardized prevalence rate of HIV-XDR-TB per 100,000 cases in 2021 by country. HIV‑XDR-TB, HIV-AIDS-extensively drug-resistant tuberculosis.

**Table S1** Incidence, deaths, prevalence, and disability-adjusted life years (DALYs) for **HIV/AIDS-Drug-susceptible Tuberculosis** in 2021 and the percentage change in the age‑standardized rates (ASRs) per 100,000 by SID and global burden of disease region, from 1990 to 2021.

|  | Incidence | |  | Deaths | |  | Prevalence | |  | DALYs | |
| --- | --- | --- | --- | --- | --- | --- | --- | --- | --- | --- | --- |
| Region | Number  (95% UI） | percentage change in age‑standardized rates (ASRs) per 100,000 (95% UI)  1990 to 2021 |  | Number  (95% UI) | percentage change in age‑standardized rates (ASRs) per 100,000 (95% UI)  1990 to 2021 |  | Number  (95% UI) | percentage change in age‑standardized rates (ASRs) per 100,000 (95% UI)  1990 to 2021 |  | Number  (95% UI) | percentage change in age‑standardized rates (ASRs) per 100,000 (95% UI)  1990 to 2021 |
| Global | 955220.60  (854660.52-1075239.94) | -0.31  (-0.40--0.19) |  | 182597.43  (141923.37-225076.20) | 0.04  (-0.22-0.47) |  | 1682114.84  (1494990.34-1881081.77) | 0.09  (0.03-0.17） |  | 9910865.77  (7825965.89-12110494.40） | -0.04  (-0.26-0.30） |
| Low-middle SDI | 256258.43  (225676.21-294178.60) | 0.93  (0.74-1.13) |  | 54499.42  (40047.85-71754.00) | 0.15  (-0.22-0.78) |  | 453519.86  (399294.25-518010.97) | -0.11  (-0.18--0.04） |  | 2916381.56  (2181803.47-3799552.55) | 0.00  (-0.30-0.45） |
| High SDI | 3386.31  (2956.41-3883.65) | 0.00  (0.00-0.00) |  | 709.70  (468.88-1058.00) | -0.86  (-0.88--0.84) |  | 4041.04  (3487.27-4668.80) | -0.77  (-0.78--0.76） |  | 30510.57  (20572.87-44782.29) | -0.87  (-0.89--0.85） |
| Middle SDI | 373579.79  (335384.32-419400.17) | -0.09  (-0.18-0.01) |  | 57666.35  (45976.57-67016.56) | 3.51  (2.80-4.31) |  | 600860.58  (536310.04-673728.33） | 2.80  (2.40-3.24) |  | 3026526.17  (2459493.45-3490842.56) | 3.19  (2.59-3.83） |
| High-middle SDI | 17695.07  (15021.38-20856.83) | -0.25  (-0.32--0.16) |  | 2923.18  (1790.18-4322.38) | -0.16  (-0.30--0.01) |  | 25894.10  (22232.69-29755.29） | 0.11  (0.02-0.23) |  | 145530.24  (93794.61-209570.64) | -0.19  (-0.31--0.06） |
| Low SDI | 303825.73  (268085.43-342864.54) | -0.73  (-0.74--0.71) |  | 66684.93  (51531.65-81943.75) | -0.56  (-0.69--0.32) |  | 596878.99  (523033.80-669614.46） | -0.51  (-0.55--0.47) |  | 3785772.08  (3010556.61-4605106.96) | -0.58  (-0.70--0.39) |
| Central Asia | 634.43  (496.72-774.59) | -0.45  (-0.49--0.40) |  | 95.82  (50.71-164.85) | -0.60  (-0.75--0.44) |  | 889.81  (694.16-1099.26） | -0.42  (-0.54--0.28) |  | 5261.68  (2918.28-8776.97) | -0.61  (-0.74--0.46) |
| Central Europe | 381.54  (328.21-437.87) | 0.33  (0.16-0.57) |  | 71.07  (47.71-103.63) | -0.56  (-0.62--0.44) |  | 476.44  (412.12-551.28） | -0.41  (-0.52--0.11) |  | 3721.34  (2577.72-5348.97) | -0.61  (-0.66--0.51) |
| East Asia | 21606.29  (18375.76-24539.72) | 0.18  (0.12-0.25) |  | 3091.67  (1847.34-4601.96) | 1.94  (0.40-21.45) |  | 43218.37  (35661.16-50080.48） | 1.60  (1.03-2.41) |  | 148308.65  (95435.15-212936.14) | 2.09  (0.59-13.88) |
| Oceania | 1196.33  (1061.71-1336.00) | -0.40  (-0.48--0.22) |  | 332.76  (235.83-485.68) | 34.11  (20.73-49.35) |  | 4095.11  (3622.97-4565.67） | 29.05  (20.68-39.56) |  | 18058.33  (13394.53-25746.57) | 30.61  (19.97-42.90) |
| Eastern Europe | 5742.88  (4122.43-7695.12) | 0.94  (0.62-1.59) |  | 674.7  (320.91-1260.34) | -0.24  (-0.59-0.14) |  | 5266.64  (3763.55-6958.51） | -0.06  (-0.30-0.17) |  | 35772.84  (17860.47-65092.81) | -0.25  (-0.57-0.12) |
| Southern Latin America | 2837.51  (2455.10-3311.79) | -0.85  (-0.85--0.84) |  | 806.69  (527.47-1023.44) | 0.53  (0.01-0.93) |  | 3695.28  (3188.89-4243.17） | -0.34  (-0.39--0.29) |  | 38189.39  (25330.58-48155.71) | 0.40  (-0.05-0.77) |
| High-income North America | 757.86  (651.38-881.81) | -0.84  (-0.85--0.83) |  | 174.86  (111.20-268.87) | -0.90  (-0.92--0.89) |  | 950.09  (802.28-1123.90） | -0.84  (-0.86--0.83) |  | 7192.73  (4714.43-10883.08) | -0.92  (-0.93--0.90) |
| Southeast Asia | 50309.00  (45212.58-55424.65) | -0.36  (-0.41--0.30) |  | 9418.85  (7338.46-11747.64) | 2.76  (2.03-3.64) |  | 93848.44  (83549.34-104117.68） | 2.26  (2.00-2.62) |  | 492619.55  (393931.03-598889.87) | 2.39  (1.81-3.12) |
| Western Sub-Saharan Africa | 116327.13  (101778.14-131446.71) | -0.09  (-0.15--0.01) |  | 27134.03  (17948.11-39119.65) | -0.10  (-0.37-0.36) |  | 233301.02  (203029.46-263919.11） | -0.31  (-0.38--0.22) |  | 1489633.26  (1022973.44-2122499.85) | -0.21  (-0.43-0.17) |
| Western Europe | 1428.35  (1240.71-1642.93) | 4.90  (4.03-5.84) |  | 413.45  (266.66-624.67) | -0.89  (-0.90--0.86) |  | 1589.94  (1362.69-1848.22） | -0.87  (-0.88--0.86) |  | 17729.77  (11688.20-26502.99) | -0.90  (-0.91--0.88) |
| Southern Sub-Saharan Africa | 331854.58  (294313.14-373263.11) | -0.47  (-0.51--0.43) |  | 52778.12  (44113.58-58185.37) | 2.05  (1.06-3.58) |  | 530816.09  (469127.05-602263.41） | 1.15  (0.93-1.43) |  | 2795866.85  (2352749.68-3089287.54) | 1.55  (0.81-2.49) |
| High-income Asia Pacific | 683.10  (587.37-785.01) | -0.47  (-0.51--0.44) |  | 114.45  (91.51-130.48) | 0.90  (0.57-1.15) |  | 494.55  (412.77-590.73） | -0.45  (-0.57-0.78) |  | 4321.58  (3526.50-4903.00) | 0.56  (0.29-0.80) |
| Central Latin America | 5919.03  (5207.12-6769.48) | -0.48  (-0.53--0.44) |  | 1549.95  (990.33-2286.37) | -0.52  (-0.62--0.38) |  | 9454.00  (8190.12-10880.91) | -0.41  (-0.47--0.35) |  | 80134.95  (52689.95-116046.79) | -0.53  (-0.62--0.40) |
| Caribbean | 2831.44  (2494.91-3193.30) | -0.34  (-0.41--0.28) |  | 863.73  (467.47-2142.88) | -0.67  (-0.75--0.47) |  | 3961.87  (3385.87-4597.62) | -0.50  (-0.54--0.46) |  | 43642.08  (24374.44-105760.09) | -0.69  (-0.77--0.51) |
| Andean Latin America | 2767.61  (2346.25-3224.05) | 14.49  (12.14-21.37) |  | 493.88  (297.39-795.02) | 0.33  (-0.19-1.15) |  | 3488.39  (2937.87-4100.96) | -0.35  (-0.46--0.23) |  | 25730.02  (15956.20-40745.74) | 0.24  (-0.22-0.94) |
| Tropical Latin America | 13833.96  (11684.09-16224.64) | -0.26  (-0.32--0.20) |  | 2520.65  (1547.07-3857.61) | -0.50  (-0.63--0.37) |  | 15171.57  (12930.13-17614.83) | -0.49  (-0.55--0.44) |  | 121927.09  (76744.46-183312.12) | -0.56  (-0.67--0.45) |
| Australasia | 42.87  (36.65-50.17) | 1.80  (1.65-1.95) |  | 4.76  (2.86-7.64) | -0.90  (-0.92--0.88) |  | 34.01  (28.69-39.95) | -0.86  (-0.87--0.85) |  | 214.70  (133.90-338.11) | -0.91  (-0.92--0.89) |
| Central Sub-Saharan Africa | 44102.11  (39222.56-49386.71) | 2.50  (2.21-2.82) |  | 9276.37  (6841.97-12279.88) | -0.54  (-0.71--0.21) |  | 97430.93  (86635.87-108993.51) | -0.51  (-0.54--0.47) |  | 530408.31  (395291.66-688591.61) | -0.57  (-0.71--0.31) |
| North Africa and Middle East | 5057.46  (4445.10-5786.80) | -0.34  (-0.47--0.21) |  | 800.64  (543.65-1115.52） | 0.53  (-0.03-1.50) |  | 5916.45  (5148.51-6779.19) | 0.39  (0.20-0.63 |  | 42087.46  (29348.37-58530.16) | 0.43  (-0.05-1.23) |
| Eastern Sub-Saharan Africa | 278559.71  (244160.44-318444.98) | -0.82  (-0.83--0.80) |  | 62078.40  (47085.85-77527.91） | -0.55  (-0.70--0.28) |  | 522224.58  (453808.20-590925.62) | -0.51  (-0.55--0.47 |  | 3493901.36  (2695620.25-4315084.04) | -0.58  (-0.72--0.37) |
| South Asia | 68347.42  (56745.06-80153.63) | -0.25  (-0.41-1.93) |  | 9902.56  (6578.30-13499.33） | 43.90  (19.26-116.44) |  | 105791.26  (88304.14-123954.41) | 5.48  (4.37-6.81) |  | 516143.85  (351094.50-697044.73) | 27.74  (14.56-50.68) |

**Table S2** Incidence, deaths, prevalence, and disability-adjusted life years (DALYs) for **HIV/AIDS-Multidrug-resistant Tuberculosis without extensive drug resistance** in 2021 and the percentage change in the age‑standardized rates (ASRs) per 100,000 by SID and global burden of disease region, from 1990 to 2021.

|  | Incidence | |  | Deaths | |  | Prevalence | |  | DALYs | |
| --- | --- | --- | --- | --- | --- | --- | --- | --- | --- | --- | --- |
| Region | Number  (95% UI） | percentage change in age‑standardized rates (ASRs) per 100,000 (95% UI)  1990 to 2021 |  | Number  (95% UI) | percentage change in age‑standardized rates (ASRs) per 100,000 (95% UI)  1990 to 2021 |  | Number  (95% UI) | percentage change in age‑standardized rates (ASRs) per 100,000 (95% UI)  1990 to 2021 |  | Number  (95% UI) | percentage change in age‑standardized rates (ASRs) per 100,000 (95% UI)  1990 to 2021 |
| Global | 45589.40  (31326.35-66723.02) | 14.44  (7.52-25.68) |  | 17457.58  (7574.38-32229.49) | 14.58  (8.31-27.16) |  | 71455.41  (48998.78-106009.43) | 15.93  (8.37-28.21) |  | 925471.28  (413529.64-1668293.09) | 14.05  (7.87-26.04) |
| Low-middle SDI | 13194.20  (7640.05-20424.31) | 19.61  (5.22-65.62) |  | 5303.90  (2160.05-10458.59) | 23.65  (7.44-78.47) |  | 20918.56  (12369.44-32076.28) | 19.31  (5.37-61.56) |  | 275699.67  (114646.24-522799.89) | 20.54  (6.17-66.69) |
| High SDI | 102.25  (64.97-167.07) | -0.63  (-0.80--0.32) |  | 37.88  (14.97-80.75) | -0.79  (-0.89--0.62) |  | 129.35  (82.78-213.47） | -0.55  (-0.75--0.17) |  | 1647.40  (683.19-3441.94) | -0.81  (-0.90--0.65) |
| Middle SDI | 14902.52  (7505.04-29959.05） | 15.54  (4.65-49.34) |  | 4883.74  (1662.98-10664.57 | 30.92  (10.80-90.70) |  | 22632.11  (11423.63-45654.37） | 20.64  (6.55-63.32) |  | 247085.88  (87758.50-536136.05) | 31.28  (11.27-88.25) |
| High-middle SDI | 3900.72  (2406.00-5908.01） | 14.91  (7.27-32.89) |  | 682.16  (343.19-1129.90 | 8.10  (3.74-19.23) |  | 3260.72  (2102.76-5153.99） | 12.75  (5.64-29.07) |  | 34557.33  (17715.91-55956.83) | 8.02  (3.68-18.58) |
| Low SDI | 13473.21  (7954.32-21578.97） | 9.97  (3.51-23.21) |  | 6542.06  (2633.61-12881.95 | 8.88  (3.21-26.21) |  | 24482.54  (14804.80-38103.60) | 9.38  (3.28-21.86) |  | 366056.18  (151659.57-725019.14) | 8.29  (2.95-24.32) |
| Central Asia | 242.64  (151.30-369.58） | 124.84  (39.91-356.20) |  | 54.82  (25.20-89.59） | 61.99  (19.38-196.68) |  | 291.41  (181.97-445.45） | 124.89  (39.17-391.81) |  | 2918.67  (1397.53-4701.00） | 62.01  (19.51-195.64) |
| Central Europe | 8.97  (3.95-18.12） | 0.60  (-0.49-4.04) |  | 3.32  (0.93-7.54） | 0.21  (-0.63-3.44) |  | 10.10  (4.65-19.67) | 1.18  (-0.27-5.47) |  | 171.48  (49.10-390.15) | 0.01  (-0.71-2.79) |
| East Asia | 1141.24  (228.83-3390.48） | 1.36  (-0.64-11.37) |  | 310.76  (50.57-942.33) | 3.01  (-0.51-78.95) |  | 2236.71  (381.26-6906.69） | 3.08  (-0.46-26.32) |  | 14108.81  (2421.93-41414.26） | 3.32  (-0.46-69.84) |
| Oceania | 54.40  (15.85-132.01） | 953.96  (195.84-3300.29) |  | 29.99  (6.69-76.08) | 1529.45  (369.49-5526.74) |  | 171.92  (49.67-407.78） | 2169.85  (487.40-7568.75) |  | 1548.33  (349.32-3947.81） | 1413.68  (350.23-5120.04) |
| Eastern Europe | 3434.58  (2084.29-5233.23） | 38.14  (14.13-94.89) |  | 555.04  (280.33-924.28) | 18.39  (6.99-52.98) |  | 2506.79  (1577.10-3672.32） | 28.29  (10.91-67.41) |  | 28659.71  (14774.00-47632.06） | 18.08  (6.85-51.79) |
| Southern Latin America | 45.71  (11.60-141.77） | 2.04  (-0.47-16.05) |  | 24.81  (4.80-83.31) | 4.51  (0.00-26.67) |  | 53.53  (13.50-175.58) | 1.73  (-0.49-13.96) |  | 1148.24  (224.98-3834.98） | 4.22  (-0.04-24.95) |
| High-income North America | 13.72  (5.43-32.98） | -0.93  (-0.97--0.77) |  | 6.33  (1.69-18.51） | -0.95  (-0.98--0.86) |  | 16.37  (6.26-41.71） | -0.92  (-0.97--0.74) |  | 253.16  (69.42-730.99） | -0.96  (-0.99--0.88) |
| Southeast Asia | 1722.56  (879.53-3019.10） | 17.68  (5.59-57.02) |  | 615.83  (227.11-1254.86） | 18.84  (4.86-77.43) |  | 3166.26  (1615.43-5525.84） | 23.46  (7.15-78.24) |  | 31412.40  (11908.13-63070.69） | 17.01  (4.32-69.27) |
| Western Sub-Saharan Africa | 4728.33  (2160.50-9841.68） | 4.86  (1.02-16.6) |  | 2327.98  (715.07-5439.05） | 4.45  (0.89-17.96) |  | 8670.73  (4120.57-17156.90） | 4.24  (0.88-14.34) |  | 124064.03  (39571.04-284092.59） | 3.77  (0.67-15.54) |
| Western Europe | 38.91  (23.52-63.11） | -0.61  (-0.81--0.21) |  | 20.93  (7.33-47.42） | -0.67  (-0.84--0.32) |  | 40.07  (24.60-62.94） | -0.56  (-0.78--0.11) |  | 877.99  (318.04-1955.44） | -0.71  (-0.86--0.41) |
| Southern Sub-Saharan Africa | 13123.40  (6235.44-28458.34 | 18.05  (4.37-79.09) |  | 4876.84  (1803.52-10740.13 | 36.31  (7.69-219.10) |  | 20032.52  (9192.34-42752.54 | 23.70  (5.27-108.91) |  | 250958.97  (94722.13-543970.49 | 30.77  (6.52-184.92) |
| High-income Asia Pacific | 10.23  (2.89-31.28） | 0.43  (-0.75-5.44) |  | 2.81  (0.64-8.79） | 2.11  (-0.28-11.14) |  | 6.16  (1.76-18.98） | 0.10  (-0.83-8.57) |  | 104.31  (24.33-320.40） | 1.67  (-0.36-9.34) |
| Central Latin America | 224.30  (95.53-452.89） | 12.91  (3.68-39.53) |  | 114.31  (33.66-246.67） | 10.41  (3.07-29.66) |  | 334.48  (135.92-693.33） | 14.83  (4.03-46.39) |  | 5748.33  (1758.44-12297.64） | 10.13  (3.05-28.52) |
| Caribbean | 16.17  (5.63-39.66） | -0.41  (-0.86-1.43) |  | 9.74  (1.93-32.05） | -0.50  (-0.89-1.20) |  | 22.28  (8.77-50.91） | -0.20  (-0.78-1.47) |  | 479.80  (98.74-1550.93） | -0.53  (-0.90-1.08) |
| Andean Latin America | 240.32  (108.27-493.10） | 5.67  (1.00-24.67) |  | 88.65  (27.86-196.46） | 10.56  (2.24-43.89) |  | 282.60  (125.30-580.84） | 5.35  (0.91-23.45) |  | 4508.63  (1454.23-9966.51） | 10.2  (2.16-41.48) |
| Tropical Latin America | 549.23  (118.27-1539.15） | 45.28  (4.77-430.61) |  | 189.74  (32.20-592.81） | 31.38  (3.49-308.34) |  | 544.74  (119.98-1563.45） | 37.95  (4.16-379.96) |  | 8950.38  (1542.74-27613.00） | 27.34  (2.95-273.50) |
| Australasia | 1.56  (0.58-3.61） | -0.02  (-0.76-3.67) |  | 0.39  (0.09-1.06） | -0.41  (-0.85-1.69) |  | 1.20  (0.46-2.81） | -0.17  (-0.79-2.83) |  | 16.98  (4.16-45.72） | -0.45  (-0.86-1.50) |
| Central Sub-Saharan Africa | 1169.45  (433.90-2581.43） | 3.21  (0.00-20.43) |  | 560.41  (167.08-1348.11） | 2.78  (-0.20-20.25） |  | 2488.51  (925.63-5599.75） | 3.17  (-0.04-20.91） |  | 31118.05  (9225.12-75702.41） | 2.54  (-0.23-19.13） |
| North Africa and Middle East | 136.72  (74.44-259.44） | 12.28  (3.97-34.63) |  | 47.79  (15.99-99.90） | 16.32  (5.39-49.57） |  | 158.11  (87.87-278.49） | 18.98  (7.13-50.98） |  | 2438.59  (829.29-5034.97） | 15.41  (5.13-45.72） |
| Eastern Sub-Saharan Africa | 12361.93  (6912.27-20663.83） | 26.38  (9.10-69.85) |  | 5925.99  (2418.74-12025.23） | 27.20  (8.43-88.72） |  | 21594.97  (12290.69-36375.37） | 25.58  (9.47-67.97） |  | 331047.86  (136235.45-659836.98） | 25.33  (8.32-79.72） |
| South Asia | 6325.06  (1714.16-14983.90） | 483.32  (54.90-5132.99） |  | 1691.13  (398.04-3936.55） | 3356.9  (493.55-42803.18） |  | 8825.96  (2377.16-21121.18） | 581.21  (63.40-7557.26） |  | 84936.55  (20924.75-200491.00） | 2653.53  (373.49-33653.19） |

**Table S3** Incidence, deaths, prevalence, and disability-adjusted life years (DALYs) for **HIV/AIDS-Extensively drug-resistant Tuberculosis** in 2021 and the percentage change in the age‑standardized rates (ASRs) per 100,000 by SID and global burden of disease region, from 1990 to 2021.

|  | Incidence | |  | Deaths | |  | Prevalence | |  | DALYs | |
| --- | --- | --- | --- | --- | --- | --- | --- | --- | --- | --- | --- |
| Region | Number  (95% UI） | percentage change in age‑standardized rates (ASRs) per 100,000 (95% UI)  1990 to 2021 |  | Number  (95% UI) | percentage change in age‑standardized rates (ASRs) per 100,000 (95% UI)  1990 to 2021 |  | Number  (95% UI) | percentage change in age‑standardized rates (ASRs) per 100,000 (95% UI)  1990 to 2021 |  | Number  (95% UI) | percentage change in age‑standardized rates (ASRs) per 100,000 (95% UI)  1990 to 2021 |
| Global | 1606.42  (1163.94-2182.83） | 0.00  (0.00-.000) |  | 840.00  (385.44-1491.72） | 0.00  (0.00-.000) |  | 1726.75  (1241.46-2426.98） | 0.00  (0.00-.000) |  | 42094.84  (19698.03-74093.39） | 0.00  (0.00-.000) |
| Low-middle SDI | 272.56  (167.34-437.47） | 0.00  (0.00-.000) |  | 171.79  (67.99-319.63） | 0.00  (0.00-.000) |  | 376.69  (216.51-621.71） | 0.00  (0.00-.000) |  | 8634.79  (3426.24-15905.72） | 0.00  (0.00-.000) |
| High SDI | 15.59  (10.58-23.26） | 0.00  (0.00-.000) |  | 9.95  (3.80-20.24） | 0.00  (0.00-.000) |  | 16.06  (10.82-24.53） | 0.00  (0.00-.000) |  | 426.39  (167.90-847.54） | 0.00  (0.00-.000) |
| Middle SDI | 401.20  (239.18-611.69） | 0.00  (0.00-.000) |  | 262.60  (92.37-537.72） | 0.00  (0.00-.000) |  | 549.52  (331.03-913.53） | 0.00  (0.00-.000) |  | 12710.18  (4630.68-25783.67） | 0.00  (0.00-.000) |
| High-middle SDI | 771.98  (477.44-1160.66） | 0.00  (0.00-.000) |  | 285.87  (142.68-480.64） | 0.00  (0.00-.000) |  | 591.23  (380.65-872.49） | 0.00  (0.00-.000) |  | 14330.61  (7295.76-23981.73） | 0.00  (0.00-.000) |
| Low SDI | 144.31  (88.74-224.95） | 0.00  (0.00-.000) |  | 109.2  (42.36-219.23） | 0.00  (0.00-.000) |  | 191.97  (116.90-295.78） | 0.00  (0.00-.000) |  | 5963.09  (2275.71-11935.74） | 0.00  (0.00-.000) |
| Central Asia | 53.74  (33.43-81.31） | 0.00  (0.00-.000) |  | 25.61  (11.74-45.13） | 0.00  (0.00-.000) |  | 61.27  (38.27-93.67） | 0.00  (0.00-.000) |  | 1332.72  (621.50-2330.74） | 0.00  (0.00-.000) |
| Central Europe | 2.26  (0.97-4.64） | 0.00  (0.00-.000) |  | 1.55  (0.44-3.58） | 0.00  (0.00-.000) |  | 2.12  (0.98-4.14） | 0.00  (0.00-.000) |  | 78.90  (22.65-183.40） | 0.00  (0.00-.000) |
| East Asia | 104.15  (20.60-311.71） | 0.00  (0.00-.000) |  | 60.74  (8.75-204.47） | 0.00  (0.00-.000) |  | 195.97  (33.40-605.09） | 0.00  (0.00-.000) |  | 2658.63  (400.05-8804.67） | 0.00  (0.00-.000) |
| Oceania | 7.30  (2.13-17.71） | 0.00  (0.00-.000) |  | 5.82  (1.22-14.92） | 0.00  (0.00-.000) |  | 15.06  (4.35-35.71） | 0.00  (0.00-.000) |  | 292.95  (62.10-748.82） | 0.00  (0.00-.000) |
| Eastern Europe | 724.35  (439.64-1103.31） | 0.00  (0.00-.000) |  | 259.41  (127.41-429.03） | 0.00  (0.00-.000) |  | 527.10  (331.72-772.38） | 0.00  (0.00-.000) |  | 13133.20  (6492.33-21603.74） | 0.00  (0.00-.000) |
| Southern Latin America | 8.56  (2.25-26.08） | 0.00  (0.00-.000) |  | 6.69  (1.29-22.88） | 0.00  (0.00-.000) |  | 6.48  (1.64-21.32） | 0.00  (0.00-.000) |  | 306.33  (59.32-1045.26） | 0.00  (0.00-.000) |
| High-income North America | 2.23  (0.87-5.50） | 0.00  (0.00-.000) |  | 1.70  (0.45-4.90） | 0.00  (0.00-.000) |  | 1.98  (0.76-5.05） | 0.00  (0.00-.000) |  | 66.92  (17.81-189.89） | 0.00  (0.00-.000) |
| Southeast Asia | 172.57  (86.26-305.22） | 0.00  (0.00-.000) |  | 120.49  (41.39-278.23） | 0.00  (0.00-.000) |  | 277.40  (141.58-484.12） | 0.00  (0.00-.000) |  | 6006.75  (2096.38-13545.88） | 0.00  (0.00-.000) |
| Western Sub-Saharan Africa | 41.96  (19.78-84.06） | 0.00  (0.00-.000) |  | 32.27  (9.59-72.81） | 0.00  (0.00-.000) |  | 54.06  (25.69-106.95） | 0.00  (0.00-.000) |  | 1692.86  (502.11-3793.15） | 0.00  (0.00-.000) |
| Western Europe | 7.52  (4.39-12.47） | 0.00  (0.00-.000) |  | 5.63  (1.98-12.03） | 0.00  (0.00-.000) |  | 4.85  (2.98-7.61） | 0.00  (0.00-.000) |  | 233.97  (83.57-491.31） | 0.00  (0.00-.000) |
| Southern Sub-Saharan Africa | 97.56  (47.99-205.83） | 0.00  (0.00-.000) |  | 67.87  (23.18-163.69） | 0.00  (0.00-.000) |  | 124.91  (57.31-266.69） | 0.00  (0.00-.000) |  | 3439.73  (1192.61-8315.79） | 0.00  (0.00-.000) |
| High-income Asia Pacific | 1.47  (0.43-4.37） | 0.00  (0.00-.000) |  | 0.75  (0.18-2.19） | 0.00  (0.00-.000) |  | 0.75  (0.21-2.30） | 0.00  (0.00-.000) |  | 27.58  (6.50-78.85） | 0.00  (0.00-.000) |
| Central Latin America | 25.16  (10.91-48.98） | 0.00  (0.00-.000) |  | 19.34  (5.85-44.62） | 0.00  (0.00-.000) |  | 25.47  (10.35-52.77） | 0.00  (0.00-.000) |  | 960.01  (296.50-2233.76） | 0.00  (0.00-.000) |
| Caribbean | 2.13  (0.70-5.44) | 0.00  (0.00-.000) |  | 1.66  (0.32-5.72） | 0.00  (0.00-.000) |  | 1.70  (0.67-3.88) | 0.00  (0.00-.000) |  | 80.81  (15.55-278.21) | 0.00  (0.00-.000) |
| Andean Latin America | 21.45  (9.80-43.74) | 0.00  (0.00-.000) |  | 15.03  (4.40-35.67） | 0.00  (0.00-.000) |  | 21.52  (9.55-44.26） | 0.00  (0.00-.000) |  | 753.61  (223.76-1785.45） | 0.00  (0.00-.000) |
| Tropical Latin America | 48.64  (11.08-135.23) | 0.00  (0.00-.000) |  | 32.11  (5.09-109.66) | 0.00  (0.00-.000) |  | 41.48  (9.13-119.07) | 0.00  (0.00-.000) |  | 1493.87  (239.70-5083.62) | 0.00  (0.00-.000) |
| Australasia | 0.19  (0.07-0.45) | 0.00  (0.00-.000) |  | 0.11  (0.02-0.29) | 0.00  (0.00-.000) |  | 0.15  (0.06-0.34) | 0.00  (0.00-.000) |  | 4.49  (1.04-12.26) | 0.00  (0.00-.000) |
| Central Sub-Saharan Africa | 10.17  (4.01-22.08) | 0.00  (0.00-.000) |  | 7.80  (2.20-21.68) | 0.00  (0.00-.000) |  | 15.52  (5.77-34.96) | 0.00  (0.00-.000) |  | 425.58  (120.51-1190.65) | 0.00  (0.00-.000) |
| North Africa and Middle East | 5.75  (3.17-10.49) | 0.00  (0.00-.000) |  | 3.63  (1.17-8.14) | 0.00  (0.00-.000) |  | 5.39  (2.99-9.51) | 0.00  (0.00-.000) |  | 182.64  (58.91-402.38) | 0.00  (0.00-.000) |
| Eastern Sub-Saharan Africa | 107.24  (60.22-180.27) | 0.00  (0.00-.000) |  | 82.32  (31.84-173.57) | 0.00  (0.00-.000) |  | 134.65  (76.66-226.82) | 0.00  (0.00-.000) |  | 4536.71  (1754.64-9556.38) | 0.00  (0.00-.000) |
| South Asia | 162.02  (45.42-382.60) | 0.00  (0.00-.000) |  | 89.45  (19.72-228.14) | 0.00  (0.00-.000) |  | 208.94  (56.28-499.86) | 0.00  (0.00-.000) |  | 4386.58  (998.92-10876.74) | 0.00  (0.00-.000) |

**Table S4** Age-standardized incidence, death, prevalence, and DALY rates in 2021 for **HIV/AIDS-Drug-susceptible Tuberculosis** by SID and global burden of disease region.

|  | Incidence |  | Deaths |  | Prevalence |  | DALYs |
| --- | --- | --- | --- | --- | --- | --- | --- |
| Region | age‑standardized rates (ASRs) per 100,000 (95% UI) |  | age‑standardized rates (ASRs) per 100,000 (95% UI) |  | age‑standardized rates (ASRs) per 100,000 (95% UI) |  | age‑standardized rates (ASRs) per 100,000 (95% UI) |
| Global | 11.59  (10.37-13.05) |  | 2.22  (1.73-2.74) |  | 20.41  (18.14-22.82) |  | 122.54  (96.79-149.60) |
| Low-middle SDI | 13.69  (12.08-15.72) |  | 2.94  (2.16-3.83) |  | 24.21  (21.32-27.66) |  | 152.62  (114.05-198.07) |
| High SDI | 0.25  (0.22-0.29) |  | 0.05  (0.03-0.08) |  | 0.30  (0.26-0.35) |  | 2.42  (1.63-3.56) |
| Middle SDI | 13.82  (12.42-15.51) |  | 2.12  (1.68-2.46) |  | 22.22  (19.82-24.89) |  | 113.35  (91.86-130.79) |
| High-middle SDI | 1.17  (0.99-1.38) |  | 0.19  (0.11-0.28) |  | 1.70  (1.46-1.96) |  | 9.86  (6.37-14.22) |
| Low SDI | 34.95  (30.88-39.44) |  | 7.63  (5.83-9.34) |  | 68.88  (60.48-77.35) |  | 395.23  (310.15-475.43) |
| Central Asia | 0.62  (0.48-0.75) |  | 0.09  (0.05-0.16) |  | 0.86  (0.67-1.07) |  | 5.12  (2.83-8.54) |
| Central Europe | 0.30  (0.26-0.35) |  | 0.06  (0.04-0.09) |  | 0.37  (0.32-0.43) |  | 3.41  (2.35-4.91) |
| East Asia | 1.21  (1.03-1.38) |  | 0.17  (0.10-0.25) |  | 2.42  (2.00-2.80) |  | 8.76  (5.63-12.50) |
| Oceania | 9.66  (8.57-10.78) |  | 2.74  (1.90-3.97) |  | 33.11  (29.29-36.89) |  | 140.37  (103.32-199.04) |
| Eastern Europe | 2.47  (1.77-3.30) |  | 0.28  (0.13-0.53) |  | 2.26  (1.62-2.99) |  | 15.47  (7.74-28.10) |
| Southern Latin America | 3.74  (3.23-4.36) |  | 1.07  (0.70-1.36) |  | 4.86  (4.19-5.58) |  | 51.69  (34.31-65.19) |
| High-income North America | 0.16  (0.14-0.19) |  | 0.04  (0.02-0.06) |  | 0.21  (0.17-0.24) |  | 1.66  (1.09-2.52) |
| Southeast Asia | 6.60  (5.93-7.27) |  | 1.22  (0.95-1.52) |  | 12.32  (10.97-13.66) |  | 64.34  (51.56-78.18) |
| Western Sub-Saharan Africa | 33.03  (28.92-37.33) |  | 7.84  (5.18-11.29) |  | 66.37  (57.78-75.03) |  | 386.45  (264.71-552.51) |
| Western Europe | 0.26  (0.22-0.29) |  | 0.08  (0.05-0.12) |  | 0.28  (0.24-0.33) |  | 3.63  (2.39-5.42) |
| Southern Sub-Saharan Africa | 415.95  (369.19-468.18) |  | 66.48  (55.76-73.54) |  | 666.44  (589.51-755.78) |  | 3400.80  (2869.16-3753.34) |
| High-income Asia Pacific | 0.28  (0.24-0.33) |  | 0.04  (0.04-0.05) |  | 0.20  (0.16-0.24) |  | 2.05  (1.68-2.32) |
| Central Latin America | 2.21  (1.95-2.53) |  | 0.58  (0.37-0.86) |  | 3.53  (3.05-4.06) |  | 30.21  ( 19.90-43.65) |
| Caribbean | 5.68  (5.01-6.41) |  | 1.73  (0.94-4.30) |  | 7.92  (6.77-9.19) |  | 89.57  (49.97-216.73) |
| Andean Latin America | 4.02  (3.41-4.69) |  | 0.73  (0.44-1.18) |  | 5.07  (4.27-5.96) |  | 37.60  (23.31-59.52) |
| Tropical Latin America | 5.40  (4.56-6.34) |  | 0.97  (0.60-1.49) |  | 5.92  (5.04-6.87) |  | 47.93  (30.18-72.05) |
| Australasia | 0.11  (0.10-0.13) |  | 0.01  (0.01-0.02) |  | 0.09  (0.07-0.10) |  | 0.62  (0.38-0.97) |
| Central Sub-Saharan Africa | 42.35  (37.64-47.42) |  | 8.92  (6.62-11.85) |  | 93.62  (83.18-104.70) |  | 461.74  (346.48-600.28) |
| North Africa and Middle East | 0.78  (0.69-0.90) |  | 0.13  (0.09-0.18) |  | 0.91  (0.79-1.04) |  | 6.44  (4.49-8.94) |
| Eastern Sub-Saharan Africa | 88.11  (77.54-101.08) |  | 19.59  (14.85-24.34) |  | 165.66  (144.49-187.93) |  | 989.91  (761.62-1210.95) |
| South Asia | 3.68  (3.06-4.32) |  | 0.54  (0.36-0.73) |  | 5.70  (4.76-6.68) |  | 27.38  (18.70-36.67) |

**Table S5** Age-standardized incidence, death, prevalence, and DALY rates in 2021for **HIV/AIDS-Multidrug-resistant Tuberculosis without extensive drug resistance** by SID and global burden of disease region.

|  | Incidence |  | Deaths |  | Prevalence |  | DALYs |
| --- | --- | --- | --- | --- | --- | --- | --- |
|  | age‑standardized rates (ASRs) per 100,000 (95% UI) |  | age‑standardized rates (ASRs) per 100,000 (95% UI) |  | age‑standardized rates (ASRs) per 100,000 (95% UI) |  | age‑standardized rates (ASRs) per 100,000 (95% UI) |
| Global | 0.55  (0.38-0.81) |  | 0.21  (0.09-0.39) |  | 0.87  (0.59-1.29) |  | 11.48  (5.13-20.78) |
| Low-middle SDI | 0.71  (0.41-1.10) |  | 0.29  (0.12-0.56) |  | 1.12  (0.66-1.72) |  | 14.40  (6.00-27.46) |
| High SDI | 0.01  (0.01-0.01) |  | 0.00  (0.00-0.01) |  | 0.01  (0.01-0.02) |  | 0.13  (0.06-0.28) |
| Middle SDI | 0.55  (0.28-1.12) |  | 0.18  (0.06-0.39) |  | 0.84  (0.42-1.69) |  | 9.29  (3.29-20.22) |
| High-middle SDI | 0.26  (0.16-0.40) |  | 0.04  (0.02-0.07) |  | 0.22  (0.14-0.34) |  | 2.35  (1.21-3.81) |
| Low SDI | 1.52  (0.90-2.41) |  | 0.73  (0.30-1.44) |  | 2.78  (1.69-4.31) |  | 37.26  (15.52-72.41) |
| Central Asia | 0.24  (0.15-0.36) |  | 0.05  (0.02-0.09) |  | 0.28  (0.18-0.43) |  | 2.84  (1.36-4.57) |
| Central Europe | 0.01  (0.00-0.02) |  | 0.00  (0.00-0.01) |  | 0.01  (0.00-0.02) |  | 0.16  (0.05-0.37) |
| East Asia | 0.06  (0.01-0.19) |  | 0.02  (0.00-0.05) |  | 0.13  (0.02-0.39) |  | 0.84  (0.14-2.44) |
| Oceania | 0.44  (0.13-1.07) |  | 0.25  (0.06-0.64) |  | 1.39  (0.40-3.30) |  | 12.07  (2.70-30.59) |
| Eastern Europe | 1.48  (0.90-2.25) |  | 0.23  (0.12-0.39) |  | 1.07  (0.67-1.57) |  | 12.38  (6.39-20.60) |
| Southern Latin America | 0.06  (0.02-0.19) |  | 0.03  (0.01-0.11) |  | 0.07  (0.02-0.23) |  | 1.55  (0.30-5.19) |
| High-income North America | 0.00  (0.00-0.01) |  | 0.00  (0.00-0.00) |  | 0.00  (0.00-0.01) |  | 0.06  (0.02-0.17) |
| Southeast Asia | 0.23  (0.12-0.40) |  | 0.08  (0.03-0.16) |  | 0.42  (0.21-0.73) |  | 4.10  (1.55-8.22) |
| Western Sub-Saharan Africa | 1.34  (0.61-2.78) |  | 0.67  (0.21-1.58) |  | 2.47  (1.17-4.88) |  | 31.97  (10.19-73.71) |
| Western Europe | 0.01  (0.00-0.01) |  | 0.00  (0.00-0.01) |  | 0.01  (0.00-0.01) |  | 0.18  (0.07-0.40) |
| Southern Sub-Saharan Africa | 16.40  (7.79-35.65) |  | 6.13  (2.27-13.47) |  | 25.07  (11.59-53.46) |  | 304.42  (114.45-658.52) |
| High-income Asia Pacific | 0.00  (0.00-0.01) |  | 0.00  (0.00-0.00) |  | 0.00  (0.00-0.01) |  | 0.05  (0.01-0.15) |
| Central Latin America | 0.08  (0.04-0.17) |  | 0.04  (0.01-0.09) |  | 0.12  (0.05-0.26) |  | 2.17  (0.66-4.62) |
| Caribbean | 0.03  (0.01-0.08) |  | 0.02  (0.00-0.06) |  | 0.04  (0.02-0.10) |  | 0.98  (0.20-3.16) |
| Andean Latin America | 0.35  (0.16-0.72) |  | 0.13  (0.04-0.29) |  | 0.41  (0.18-0.84) |  | 6.59  (2.13-14.58) |
| Tropical Latin America | 0.22  (0.05-0.60) |  | 0.07  (0.01-0.23) |  | 0.21  (0.05-0.61) |  | 3.52  (0.61-10.86) |
| Australasia | 0.00  (0.00-0.01) |  | 0.00  (0.00-0.00) |  | 0.00  (0.00-0.01) |  | 0.05  (0.01-0.13) |
| Central Sub-Saharan Africa | 1.11  (0.41-2.42) |  | 0.53  (0.16-1.27) |  | 2.38  (0.89-5.40) |  | 26.72  (8.06-64.04) |
| North Africa and Middle East | 0.02  (0.01-0.04) |  | 0.01  (0.00-0.02) |  | 0.02  (0.01-0.04) |  | 0.37  (0.13-0.78) |
| Eastern Sub-Saharan Africa | 3.82  (2.16-6.26) |  | 1.81  (0.74-3.72) |  | 6.70  (3.88-11.16) |  | 90.59  (37.58-182.99) |
| South Asia | 0.34  (0.09-0.81) |  | 0.09  (0.02-0.21) |  | 0.48  (0.13-1.14) |  | 4.50  (1.10-10.59) |

**Table S6** Age-standardized incidence, death, prevalence, and DALY rates in 2021 for **HIV/AIDS-Extensively drug-resistant Tuberculosis** by SID and global burden of disease region.

|  | Incidence |  | Deaths |  | Prevalence |  | DALYs |
| --- | --- | --- | --- | --- | --- | --- | --- |
|  | age‑standardized rates (ASRs) per 100,000 (95% UI) |  | age‑standardized rates (ASRs) per 100,00  0 (95% UI) |  | age‑standardized rates (ASRs) per 100,000  (95% UI) |  | age‑standardized rates (ASRs) per 100,000  (95% UI) |
| Global | 0.01  (0.01-0.03) |  | 0.01  (0.00-0.02) |  | 0.02  (0.02-0.03) |  | 0.51  (0.24-0.91) |
| Low-middle SDI | 0.00  (0.01-0.02) |  | 0.01  (0.00-0.02) |  | 0.02  (0.01-0.03) |  | 0.45  (0.18-0.83) |
| High SDI | 0.00  (0.00-0.00) |  | 0.00  (0.00-0.00) |  | 0.00  (0.00-0.00) |  | 0.03  (0.01-0.07) |
| Middle SDI | 0.02  (0.01-0.02) |  | 0.01  (0.00-0.02) |  | 0.02  (0.01-0.03) |  | 0.47  (0.17-0.96) |
| High-middle SDI | 0.03  (0.03-0.08) |  | 0.02  (0.01-0.03) |  | 0.04  (0.03-0.06) |  | 0.98  (0.50-1.63) |
| Low SDI | 0.01  (0.01-0.03) |  | 0.01  (0.00-0.02) |  | 0.02  (0.01-0.03) |  | 0.61  (0.24-1.22) |
| Central Asia | 0.00  (0.03-0.08) |  | 0.02  (0.01-0.04) |  | 0.06  (0.04-0.09) |  | 1.30  (0.60-2.27) |
| Central Europe | 0.19  (0.00-0.00) |  | 0.00  (0.00-0.00) |  | 0.00  (0.00-0.00) |  | 0.07  (0.02-0.18) |
| East Asia | 0.00  (0.00-0.02) |  | 0.00  (0.00-0.01) |  | 0.01  (0.00-0.03) |  | 0.16  (0.02-0.52) |
| Oceania | 0.00  (0.02-0.15) |  | 0.05  (0.01-0.12) |  | 0.12  (0.04-0.29) |  | 2.28  (0.48-5.79) |
| Eastern Europe | 0.00  (0.19-0.48) |  | 0.11  (0.05-0.18) |  | 0.23  (0.14-0.33) |  | 5.67  (2.81-9.34) |
| Southern Latin America | 0.00  (0.00-0.03) |  | 0.01  (0.00-0.03) |  | 0.01  (0.00-0.03) |  | 0.41  (0.08-1.42) |
| High-income North America | 0.00  (0.00-0.00) |  | 0.00  (0.00-0.00) |  | 0.00  (0.00-0.00) |  | 0.02  (0.00-0.04) |
| Southeast Asia | 0.00  (0.01-0.04) |  | 0.02  (0.01-0.04) |  | 0.04  (0.02-0.06) |  | 0.78  (0.27-1.76) |
| Western Sub-Saharan Africa | 0.00  (0.01-0.02) |  | 0.01  (0.00-0.02) |  | 0.02  (0.01-0.03) |  | 0.44  (0.13-0.98) |
| Western Europe | 0.00  (0.00-0.00) |  | 0.00  (0.00-0.00) |  | 0.00  (0.00-0.00) |  | 0.05  (0.02-0.10) |
| Southern Sub-Saharan Africa | 0.02  (0.06-0.26) |  | 0.09  (0.03-0.21) |  | 0.16  (0.07-0.33) |  | 4.17  (1.46-10.03) |
| High-income Asia Pacific | 0.01  (0.00-0.00) |  | 0.00  (0.00-0.00) |  | 0.00  (0.00-0.00) |  | 0.01  (0.00-0.04) |
| Central Latin America | 0.00  (0.00-0.02) |  | 0.01  (0.00-0.02) |  | 0.01  (0.00-0.02) |  | 0.36  (0.11-0.84) |
| Caribbean | 0.03  (0.00-0.01) |  | 0.00  (0.00-0.01) |  | 0.00  (0.00-0.01) |  | 0.17  (0.03-0.58) |
| Andean Latin America | 0.00  (0.01-0.06) |  | 0.02  (0.01-0.05) |  | 0.03  (0.01-0.06) |  | 1.10  (0.33-2.61) |
| Tropical Latin America | 0.01  (0.00-0.05) |  | 0.01  (0.00-0.04) |  | 0.02  (0.00-0.05) |  | 0.59  (0.09-2.00) |
| Australasia | 0.00  (0.00-0.00) |  | 0.00  (0.00-0.00) |  | 0.00  (0.00-0.00) |  | 0.01  (0.00-0.04) |
| Central Sub-Saharan Africa | 0.01  (0.00-0.02) |  | 0.01  (0.00-0.02) |  | 0.01  (0.01-0.03) |  | 0.36  (0.10-1.01) |
| North Africa and Middle East | 0.01  (0.00-0.00) |  | 0.00  (0.00-0.00)) |  | 0.00  (0.00-0.00) |  | 0.03  (0.01-0.06) |
| Eastern Sub-Saharan Africa | 0.06  (0.02-0.05) |  | 0.03  (0.01-0.05) |  | 0.04  (0.02-0.07) |  | 1.24  (0.49-2.60) |
| South Asia | 0.01  (0.00-0.02) |  | 0.00  (0.00-0.01) |  | 0.01  (0.00-0.03) |  | 0.23  (0.05-0.58) |

Table S7 The Bayesian Age-Period-Cohort (BAPC) model predicts values for 2021-2035

| Year | HIV-DS-TB | HIV-MDR-TB | HIV-XDR-TB |
| --- | --- | --- | --- |
|  | age-standardized DALY rate (95% CI) | age-standardized DALY rate (95% CI) | age-standardized DALY rate (95% CI) |
| 2022 | 145.92  (105.59-186.24) | 15.04  (8.04-22.04) | 0.72  (-0.46-1.90) |
| 2023 | 149.54  (96.00-203.07) | 16.82  (5.96-27.69) | 0.91  (-1.22-3.04) |
| 2024 | 153.46  (87.82-219.09) | 18.84  (3.90-33.79) | 1.15  (-2.18-4.48) |
| 2025 | 157.69  (80.32-235.05) | 21.14  (1.62-40.65) | 1.46  (-3.45-6.36) |
| 2026 | 162.21  (73.14-251.29) | 23.74  (-1.01-48.50) | 1.85  (-5.17-8.86) |
| 2027 | 166.94  (66.03-267.85) | 26.69  (-4.13-57.50) | 2.34  (-7.50-12.18) |
| 2028 | 171.63  (58.81-284.44) | 29.97  (-7.83-67.78) | 2.97  (-10.64-16.59) |
| 2029 | 176.16  (51.39-300.93) | 33.62  (-12.22-79.46) | 3.77  (-14.87-22.42) |
| 2030 | 180.51  (43.74-317.28) | 37.65  (-17.42-92.71) | 4.78  (-20.53-30.10) |
| 2031 | 184.64  (35.84-333.45) | 42.08  (-23.52-107.69) | 6.07  (-28.08-40.22) |
| 2032 | 188.47  (27.69-349.25) | 46.94  (-30.66-124.54) | 7.70  (-38.14-53.54) |
| 2033 | 191.73  (19.29-364.17) | 52.19  (-38.91-143.29) | 9.76  (-51.41-70.92) |
| 2034 | 194.30  (10.71-377.89) | 57.81  (-48.34-163.96) | 12.33  (-68.81-93.47) |
| 2035 | 196.18  (2.02-390.33) | 63.8  (-59.04-186.64) | 15.55  (-91.52-122.62) |
